# Insulin resistance alters cortical inhibitory neurons and microglia to exacerbate Alzheimer’s knock-in mouse phenotypes

**DOI:** 10.1186/s13024-026-00946-0

**Published:** 2026-06-03

**Authors:** LaShae Nicholson, Si Jie Tang, Tejaswini Karra, Habiba Abouelatta, Stephen M. Strittmatter

**Affiliations:** 1 Departments of Neuroscience and Neurology, Yale School of Medicine, New Haven, CT USA; 2 Program in Cellular Neuroscience, Neurodegeneration, and Repair, Yale School of Medicine, New Haven, CT USA; 3 Present Address: School of Medicine, University of California Davis Medical Center, Sacramento, CA USA; 4 Present Address: Institute of Neuropathology, University Medical Centre Göttingen, 37077 Göttingen, Germany; 5 Present Address: Department of Neuroscience and Behavior, Mount Holyoke College, South Hadley, MA USA

**Keywords:** Insulin resistance, Obesity, Glucose, Diabetes, Alzheimer’s, Microglia, Inhibitory neurons, snRNA-seq

## Abstract

**Background:**

Metabolic dysfunction contributes to the risk and progression of Alzheimer’s disease (AD), yet the cellular mechanisms linking impaired insulin signaling and systemic metabolic stress to brain dysfunction remain incompletely defined.

**Methods:**

We examined the impact of chronic high-fat, high-sugar (HFHS)-induced insulin resistance on metabolic parameters, spatial learning and memory, and in vivo glial activation and neuropathology in Alzheimer’s disease knock-in mice expressing human mutant APP and wild-type (WT) tau. Single-nucleus RNA sequencing was performed to resolve cell-type-specific transcriptional responses.

**Results:**

HFHS-diet induced weight gain, hyperglycemia, and glucose intolerance in WT and AD knock-in mice as compared to control diet-fed mice. However, impaired spatial learning was observed only in AD knock-in mice on the HFHS diet, even though there was no greater amyloid-β deposition or tau phosphorylation than in control diet AD knock-in mice. Transcriptomic profiling revealed that HFHS-fed AD mice engaged a distinct glial program, which we termed the metabolic impairment in neurodegeneration (MinD) state, characterized by upregulation of genes involved in synaptic targeting and trans-synaptic signaling shared across microglia, astrocytes, and oligodendrocytes. In parallel, we identified selective induction of the transcription factor *Meis2* in cortical Layer 2 inhibitory neurons, which exhibited HFHS-diet transcriptional remodeling enriched for pathways regulating vesicle release, synaptic organization, and membrane excitability. These coordinated glial and neuronal transcriptional changes were associated with reduced inhibitory synapse density in HFHS-fed AD mice.

**Conclusion:**

Diet-induced insulin resistance in AD knock-in mice is associated with coordinated glial and inhibitory neuron transcriptional remodeling and cognitive impairment, without alteration of the classical amyloid and tau pathology present in the AD mice fed a lean diet. These findings define cellular programs linking systemic insulin metabolic dysfunction to cortical circuity vulnerability in AD.

**Supplementary Information:**

The online version contains supplementary material available at 10.1186/s13024-026-00946-0.

## Background

Dysregulation in glucose metabolism has been implicated in Alzheimer’s disease (AD) risk and progression of cognitive decline [1–3]. Large-scale epidemiological studies support this mechanistic link, showing that even modest elevations in blood glucose are predictive of increased AD risk and accelerated cognitive decline with aging [1]. These observations implicate systemic metabolic dysfunction as a significant contributor to brain vulnerability in neurodegeneration. Impaired insulin signaling, as observed in diet-induced insulin resistance, has been linked to both metabolic and neurodegenerative disorders [4–6]. This phenomenon has sparked interest in insulin-modulating therapies, including GLP-1 receptor agonists and intranasal insulin for AD [7–9]. However, the cellular mechanisms linking systemic metabolic dysfunction to neurodegeneration remain poorly understood.

To examine how diet-induced insulin resistance interacts with the pathological progression of AD, we employed a chronic high-fat, high-sugar (HFHS) diet [10, 11] to induce metabolic dysfunction in double knock-in (DKI) Alzheimer’s mice expressing human mutant APP and human wild-type (WT) tau [12–16]. Prior studies investigating metabolic dysfunction in AD models have relied on APP or tau overexpression systems or combined metabolic insults [17, 18]. These approaches, along with variability in genetic background, age, and induction paradigms for metabolic impairment, have produced inconsistent results, making it difficult to isolate the specific contributions of metabolic stress to AD-related phenotypes [19]. By applying a chronic dietary challenge to genetically defined knock-in models with pathophysiologically relevant mutations, we sought to determine how systemic metabolic stress is associated with the behavioral changes, glial activation states, and cellular transcriptional responses in the AD brain.

To evaluate metabolic, behavioral, and cellular outcomes, we combined glucose and weight monitoring, spatial memory testing, histological analyses of glial reactivity and synaptic markers, and single-nucleus RNA sequencing (snRNA-seq) to resolve cell-type-specific transcriptional responses. Chronic HFHS-diet induced metabolic stress associated with insulin resistance in both WT and DKI mice. Spatial learning and memory deficits were pronounced only in HFHS-fed DKI mice, but not in lean-diet-fed DKI or HFHS-fed WT mice. Transcriptomic analyses identified glial cell populations and cortical inhibitory neurons as being particularly responsive to diet-induced metabolic stress. We identified and defined a distinct pan-glial transcriptional program, which we term the *metabolic impairment in neurodegeneration* (MinD) state. In parallel, the transcription factor Meis2 was selectively upregulated in cortical Layer 2 (L2) inhibitory neurons, with diet-induced changes observed in WT mice that were further amplified in DKI mice. These coordinated glial and neuronal transcriptional changes occurred without detectable alterations in amyloid-β (Aβ) or tau deposition.

Our findings indicate that diet-induced metabolic dysfunction worsens AD-related phenotypes independent of classical Aβ and tau pathology, highlighting a cellular link between systemic metabolic states and cognitive vulnerability.

## Methods

### Animal handling and husbandry

Studies were approved by Yale University’s Institutional Animal Care and Use Committee (IACUC, protocol #07281). Mice were cared for by the Yale Animal Resources Center (YARC) and housed in groups of one to five mice per cage under a 12-hour light/dark cycle, with food and water provided *ad libitum*. Homozygous *App*^*NL−G−F*^*/Mapt*^*hMAPT*^ double knock-in (DKI) and age-matched WT mice were used for this diet-induced insulin resistance study. DKI mice were generated by crossbreeding homozygous *App*^*NL−G−F/NL−G−F*^ and homozygous *Mapt*^*hMAPT/hMAPT*^ single knock-in mice, as previously described [15, 16]. Sex was balanced across all groups, with 45–55% of each sex in each experiment. Single knock-in *App*^*NL−G−F/NL−G−F*^ and *Mapt*^hMAPT/hMAPT^ mice were generously provided by Drs. Saito and Saido from the RIKEN Center for Brain Science. All mouse lines used were maintained on a C57BL/6J background after more than nine generations of backcrossing.

### HFHS diet paradigm and metabolic assessment

To induce insulin resistance, mice were placed on a chronic high-fat-high-sugar (HFHS) diet, a well-established model of diet-induced glucose impairment [10]. DKI and WT mice were randomized into age- and sex-balanced groups (*n* *= 15–20 mice* per group), initially fed *ad libitum* on a standard chow diet (Tekland Global 18% protein diet, S2018). At approximately 3–4 months of age, mice were switched to a lean diet (Research Diets, D12450K; diet composition: 20% kcal protein, 10% kcal fat, and 70% kcal carbohydrate, including 0 gm sucrose/kg chow) or HFHS diet (Research Diets, D12492; diet composition: 20% kcal protein, 60% kcal fat, and 20% kcal carbohydrate, including 88 gm sucrose/kg chow). Mice were maintained on their respective diets until the date of sacrifice.

Mouse body weight and fasting blood glucose levels were measured to assess metabolic status, with measurements taken at approximately 3-week intervals after diet onset. Blood glucose levels were measured via the tail vein, after 6 h of fasting, using the OneTouch Basic (LifeScan, Inc., USA) blood glucose monitoring system. Fasting glucose levels of 100–140 mg/dL were considered normal, whereas values exceeding 150 mg/dL were classified as chronically hyperglycemic, in accordance with established guidelines [11, 20, 21]. An oral glucose tolerance test was used to assess the acute metabolic response to glucose [11, 21]. Mice were fasted for 6 h and then administered a bolus of 20% dextrose solution (2 g/kg body weight) via oral gavage. Blood glucose levels measured 1 min before and 30 min after dextrose administration were used to compare metabolic impairment between groups.

### Morris Water Maze

Mice were evaluated at 10 months of age. Spatial learning and memory were assessed using the Morris Water Maze (MWM), conducted as previously described with slight modifications [13, 15, 16]. A 1 m-diameter pool, surrounded by visual cues, was filled with water and maintained at a temperature between 21 and 23 °C. An invisible platform was submerged 1.5 cm underwater in the target quadrant. All visual cues, water temperature, and ambient lighting were kept constant throughout the experiment. Mice underwent 4 days of habituation training prior to MWM testing. Experimental groups were randomized, and experimenters blinded to the genotype and diet conditions during behavioral acquisition and analysis. Testing occurred over 8 days: 3 days of forward training and 1 probe trial day for the forward acquisition paradigm, immediately followed by 3 days of reverse training and 1 probe day for the memory retrieval paradigm. Each acquisition training consisted of 8 trials (4 per session, conducted twice daily). Mice were released from one of four pseudo-randomized entry locations, facing away from the pool’s center and opposite the target quadrant. Entry locations were varied for each mouse in each training session. Each trial lasted up to 60 s, and success was defined as the mouse locating and remaining on the platform for at least 1 s. In the initial training session, if a mouse failed to find the platform within the time limit, it was guided backwards to the invisible platform and allowed to rest there for 10 s before being removed from the pool. The probe trial took place 24 h after the final training session, during which the transparent platform was removed, and mice were placed facing the wall opposite the target quadrant and allowed to swim for 60 s. The reverse training sessions and probe trial followed the same protocol, but the transparent platform was placed in the opposite quadrant. Mouse latencies to the platform were recorded using a JVC Everio G-series camcorder (JVCKenwood, Yokohama, Japan) and tracked by Panlab’s Smart software (Harvard Bioscience, Massachusetts, USA). Following the final probe trial, mice were assessed for visual acuity deficits. A visible platform was positioned in the target quadrant, and trials continued until latencies stabilized over a maximum of 15 trials, with average latencies calculated for the final three trials.

### Immunolabeling of mouse tissue

Mice were euthanized with CO₂ and perfused transcardially with ice-cold phosphate-buffered saline (PBS, pH 7.4), followed by ice-cold 4% paraformaldehyde (PFA) in PBS. Brains were rapidly dissected, bisected at the midline, and post-fixed in 4% PFA at 4 °C for 24 h. Post-fixed brains were sectioned into 50 μm coronal slices using a Leica VT1000S vibratome (Leica, Wetzlar, Germany). Free-floating sections were stored long-term at 4 °C in PBS containing 0.05% sodium azide. For immunofluorescence labeling, free-floating sections were incubated at room temperature for 1 h in blocking buffer (5% bovine serum albumin, 1% Triton-X, pH 7.4 PBS), then incubated overnight at 4 °C in primary antibodies diluted in blocking buffer.

The following primary antibodies were used: rabbit anti-β amyloid (1:600, RRID: AB_2797642) for Aβ deposition; rabbit anti-IBA1 (1:600, RRID: AB_839504), goat anti-IBA1 (1:500, RRID: AB_10982846), rat anti-Cd68 (1:250, RRID: AB_322219) sheep anti-Trem2 (1:125, RRID: AB_356109) for assessment of microgliosis; chicken anti-GFAP (1:1000, RRID: AB_304558) for astrocyte gliosis; rabbit anti-p(Y128)ErbB4 (1:200, RRID: AB_940986), guinea pig anti-Gephyrin (1:200, RRID: AB_2661777), guinea pig anti-Homer (1:200, RRID: AB_2661777), rabbit anti-PSD-95 (1:300, RRID: AB_87705), chicken anti-Synapsin½ (1:250, RRID: AB_2622240), rabbit anti-VGAT (1:200, RRID: AB_887871), and rabbit anti-VGLUT1 (1:200, RRID: AB_887877) for synaptic density measurements; rabbit anti-Meis2 (1:250, RRID: AB_11026529) or rabbit anti-PBX1 (1:250, RRID: AB_11205760) co-labeled with chicken anti-NeuN (1:400, RRID: AB_11205760) for the assessment of Layer 2 (L2) inhibitory neurons; guinea pig anti-Pvalb (1:700, RRID: AB_2156476), WFA-FITC (1:2000, Vector Laboratories, FL-1351-2), mouse biotinylated AT8 (1:100, RRID: AB_223648), and rabbit anti-pThr217 (1:200, RRID: AB_2533741) for assessment of Tau pathology. For optimal immunodetection of AT8, the Alexa Fluor™ 488 Tyramide SuperBoost Kit, Streptavidin (Invitrogen, B40932), was used following the manufacturer’s protocols. For pThr217 staining, an antigen retrieval step was performed prior to exposure to primary antibody by incubating slices in 1x Reveal Decloaker buffer (RV 1000 M, Biocare Medical) for 15 min at 90 °C, followed by 15 min at room temperature.

Sections were washed 3 times for 10 min each in blocking buffer and incubated in Alexa Fluor™ secondary antibodies diluted in blocking buffer (donkey or goat anti-chicken, anti-goat, anti-guinea pig, anti-rabbit, anti-rat, 1:200, ThermoFisher Scientific) for one hour at room temperature in the dark. To reduce background autofluorescence, sections were washed three times in PBS, briefly dipped in ddH_2_O, and incubated for one minute in CuSO_4_ (10 mM CuSO_4_ in 50 mM ammonium acetate buffer, pH 5.0) followed by a second brief dip in ddH_2_O. Sections received a final series of three PBS washes for 10 min, then were mounted onto Superfrost Plus microscope slides (ThermoFisher Scientific, 22-037-246) and coverslipped with Prolong™ Glass mounting medium (ThermoFisher Scientific, P36984).

### Imaging and analysis of mouse tissues

Image acquisition, reconstruction, and quantification were performed by experimenters blinded to mouse genotype and dietary conditions. All images were acquired using the Stellaris STED 8 confocal microscope (Leica Microsystems, Wetzlar, Germany). Tissues immunolabeled to assess for Aβ deposition, microgliosis (Cd68, Iba1, Trem2), astrogliosis (GFAP), tau pathology (AT8, pThr217), perineuronal nets (Pvalb and WFA), and the expression of Meis2 in NeuN^+^ cells, were imaged with a 20X, 0.8NA air objective. Images were acquired as z-stacks with a 2 μm step size within the motor and somatosensory cortices, specifically targeting L2-3, unless otherwise noted. All images were analyzed as maximum intensity projections, and the percent area occupied by threshold immunolabeled signal was quantified using ImageJ (v2.16.0, License GPLv3+). For Meis2^+^ cell quantification, binary images were generated from NeuN and Meis2 fluorescence channels. A NeuN-based mask was used to identify neuronal nuclei, and binary Meis2 + signals within this mask were isolated using *the Image Calculator* function in ImageJ. The number of Meis2 + cells was quantified using *Analyze Particles* and expressed as a percentage of total NeuN+ cells. Quantification was performed within a 200 × 500 μm region of cortical L2-3, using single z-stack planes averaged across all planes in the stack. For all quantitative analyses, two sections per mouse were imaged, analyzed, and averaged to generate a single biological replicate per animal. All statistical analyses were performed at the mouse level. Microglial morphology and Aβ plaque association were assessed from IBA1 and Aβ co-immunolabeled images acquired within cortical L2-3. Full-depth z-stacks were acquired using a 40X, 1.3 NA oil-immersion objective with 2X digital zoom and 1 μm step size. Between 5 and 6 regions per section were imaged across 2 sections per mouse. Three-dimensional (3D) reconstructions and quantitative analyses were performed using Imaris software (v10.2, Bitplane). To quantify Aβ plaque volume and microglia–plaque associations, 3D volumes were generated from the Iba1 and Aβ channels using the *Surfaces* tool. Aβ plaques were defined as objects larger than 5 μm³. Using the *object-to-object* statistics feature, the number of Iba1^+^ microglial surfaces in direct contact with Aβ plaques was quantified and reported as a proportion of total microglia per image. For microglial Sholl analysis, the *Mask* tool was used to select and isolate the Iba1^+^ signal within the volume. Then, the *filament* tool was used to trace microglial processes within the masked region for 3D morphological reconstruction and Sholl quantification. Between 4 and 6 microglial cells were analyzed per mouse. For all morphological analyses, values from individual cells were averaged to generate a single biological replicate per animal. Statistical analyses were performed at the individual-mouse level. For synaptic density quantifications, synaptic proteins were labeled with STED-compatible secondary antibodies: Alexa Fluor™ 488 and Alexa Fluor^TM^ 555 (1:200, ThermoFisher Scientific). Z-stack images were acquired within the cortical L2-3 region using a 100X, 1.4 NA oil-immersion objective with 2X digital zoom and a 0.5 μm step size. Dual-channel imaging was performed using a pulsed White Light Laser for excitation in conjunction with a 660-nm depletion laser. 3D image stacks were analyzed using Imaris. Pre- and post-synaptic puncta were detected using the *Spots* tool, and synaptic loci were defined by colocalization of puncta signals within 0.4 μm of each other in 3D space. Three regions of interest were imaged per mouse, from a single tissue section, and the mean per animal was used for statistical analyses.

### Single-nuclei RNA sequencing and analysis

Mice were sacrificed at 11.5 months by rapid decapitation. Brains were harvested and medially dissected on ice. Brain hemispheres were micro-dissected to isolate the cortex and hippocampus, then immediately frozen on dry ice and stored at −80°C. For single-nucleus RNA sequencing experiments, cortical and hippocampal tissues from the left-brain hemisphere of a single mouse were pooled. Four biological replicates per group were processed separately for sequencing. Preparation of samples, cDNA library construction, and sequencing were performed as previously described [16, 22]. Briefly, pooled cortical and hippocampal tissues were gently homogenized on ice in a buffered solution (10 mM HEPES, pH 7.9; 25 mM KCl; 1 mM EDTA; 10% glycerol; 2 M sucrose) supplemented with 80 U/mL RNase inhibitor (Roche 03335402001) to release cellular nuclei. Samples were centrifuged at 20,000 x g at 4°C for 1 hour, supernatants were discarded, and pelleted nuclei were resuspended to a concentration of 700–1200 nuclei/mL in chilled PBS supplemented with 80 U/mL RNase Inhibitor. Nuclei barcoded cDNA libraries were constructed using the Chromium Single Cell 3’ Reagents Kit v3 (10x Genomics) following the manufacturer’s guidelines. Sample libraries were pooled and collectively sequenced on an Illumina NovaSeq 5000 using single-indexed paired-end HiSeq sequencing, yielding a mean sample sequencing depth of 450 million reads and 30,000 UMIs per nucleus. Raw sequencing reads were mapped against the mm10-2020-A mouse reference gene using the Cell Ranger Count pipeline (v6.1.2, 10x Genomics) with default parameters.

Processing of snRNA-seq data was conducted as previously described, with modifications [16]. Sample gene count matrices were merged into a single AnnData object and processed using the Scanpy v1.9.6 Python package [23]. Quality control filtering removed nuclei with more than 5% of transcripts mapping to mitochondrial genes, fewer than 50 detected genes, or more than 5,000 genes. Genes expressed in fewer than 10 nuclei were also excluded. Clustering was performed in two stages. First, unsupervised clustering was conducted using Scanpy for the unbiased identification and removal of putative doublets. Gene expression data were normalized and log1p-transformed. Highly variable genes were identified using *scanpy.pp.highly_variable_genes* with *n_top_genes = 2000* and *flavor=’seurat_v3’*. Principal component analysis of variable genes was computed with *scanpy.tl.pca*, specified with *svd_solver=’arpack’*. Using the top 37 principal components, a neighborhood graph was constructed using scanpy.pp.neighbors, followed by functions *scanpy.tl.leiden* for Leiden Clustering [24] and *scanpy.tl.umap* for UMAP embedding. Cluster identities were initially assigned based on marker genes identified in our previous studies [16, 22]. Doublets, defined as individual clusters enriched for marker genes from multiple cell types, were filtered from the dataset.

Following doublet removal, downstream analyses were performed using the *model.SCVI* (scvi-tools v0.20.3) [25]. The model was trained on the filtered dataset using total gene counts and percent mitochondrial content as covariates. The latent representation from the trained scVI model was then used for batch-corrected dimensionality reduction, clustering, and visualization in UMAP space. Cluster classification and subclass annotation were based on the union of known marker gene expression profiles (Supplementary Table 1) and alignment to the Allen Brain Cell Atlas using the MapMyCells tool (RRID: SCR_024672, Supplementary Table 2).

To achieve higher-resolution analysis, specific cell populations (microglia, excitatory neurons, inhibitory neurons) were subset from the full dataset and re-analyzed independently. For each subset, a new scVI model was trained *de novo* on the cell-type-restricted AnnData object to account for population-specific variation. The latent space inferred from the scVI model was then used to perform batch-corrected reclustering and visualization.

### Differential expression and pathway enrichment analysis

Differential expression gene (DEG) analysis was performed using the *scanpy.tl.rank_genes_groups* function with the Wilcoxon rank-sum test to compare experimental groups within each identified cell type. DEGs were defined as those expressed in at least 10% of nuclei tested, with an adjusted *P*-value (Benjamini-Hochberg correction) less than 0.05, and an absolute log-transformed fold change greater than 0.25. Gene pathway enrichment and cellular compartment analysis were conducted using Cytoscape (v3.9.1) [26] with the ClueGO (v2.5.9) [27] and STRING (v2.0.0) [28] plugins. Ingenuity Pathway Analysis (IPA, Qiagen) was used to determine the subcellular localization of DEGs. For microglia, additional gene set enrichment analyses were performed using curated published cell-state signatures, including DAM and related activation states.

### Statistics and reproducibility

No statistical methods were used to predetermine sample sizes, but all experiments were conducted with comparable sample sizes. Data collection and downstream analyses were performed by experimentalists blinded to experimental conditions, and automated where possible to minimize experimenter bias. Data were analyzed using GraphPad Prism v9.2.0. Experimental analyses involving more than two groups was evaluated using two-way ANOVA, followed by appropriate multiple-comparison tests. Normality tests were performed to determine whether of parametric or non-parametric methods were appropriate. Unless indicated otherwise, data are presented as mean ± SEM, with individual data points representing intra-mouse means. Statistical details, including the exact number (*n*) of biological replicates (animals or cells), numerical means, statistical tests, and multiple comparison corrections, are provided in the figure legends. Statistical significance is denoted as *p < 0.0332, **p < 0.0021, ***p < 0.0002, and ****p < 0.0001.

## Results

### HFHS-induced metabolic impairment drives cognitive decline

**Fig. 1:**
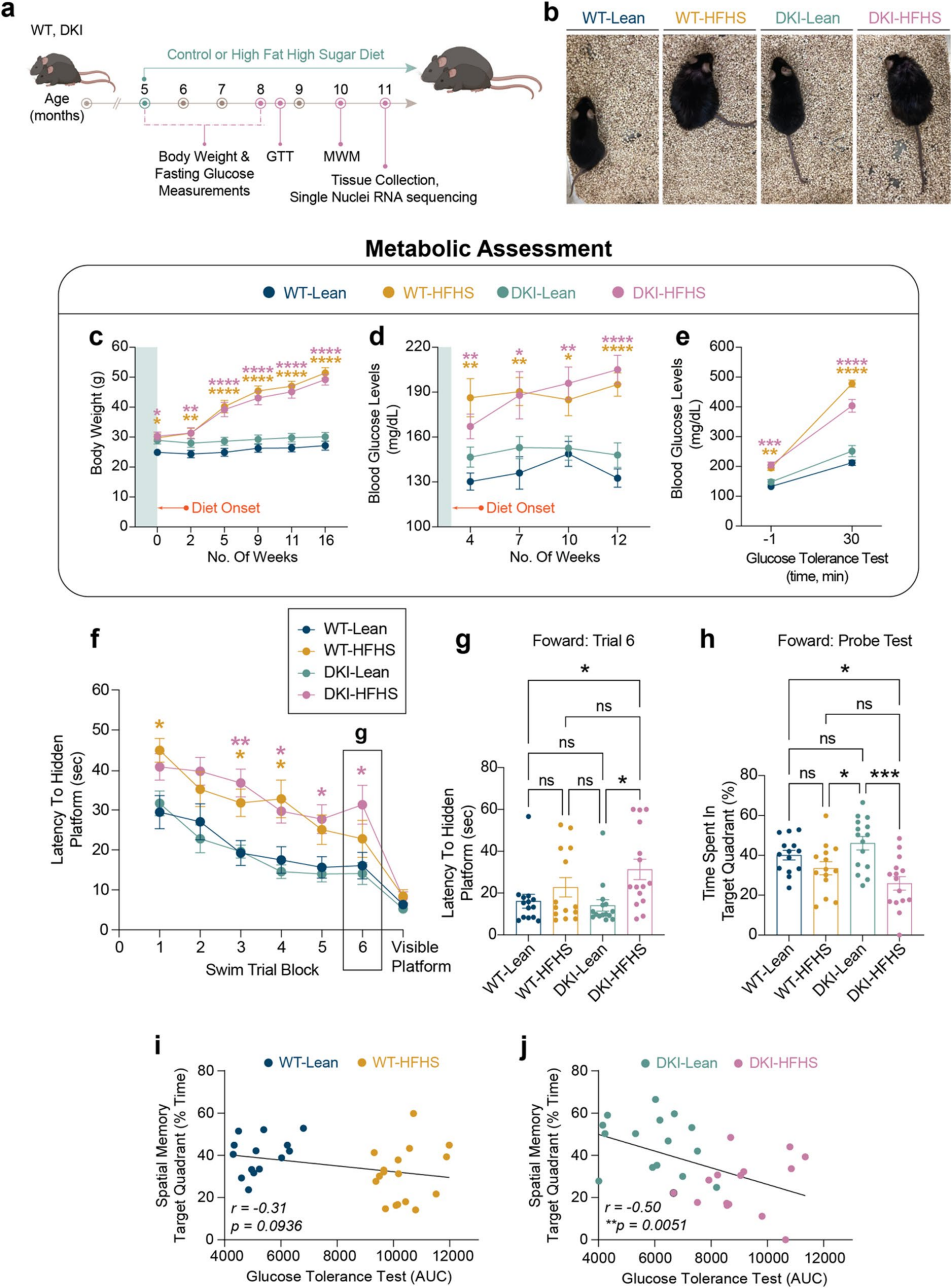
AD phenotype and HFHS diet-induced glucose intolerance synergistically impair spatial learning in DKI mice. **a**-**c**, Overview and metabolic assessment of HFHS diet exposure. **a**, Schematic of experimental design showing timepoints for behavioral and metabolic assessments. **b**, Representative images of mouse body size differences between groups. **c**, Body weight **d**, blood glucose levels **e**, and glucose intolerance test 30 min post oral glucose bolus delivery. **f**-**h**, MWM behavioral testing. **f-g**, Latency time to the hidden platform during the training phase (**f**) and final training session (**g**). **h**, Fraction of time spent in the target quadrant, during probe trial. **c**-**h**, Data are presented as the mean ± SEM. **c**-**f**, Data was analyzed by two-way ANOVA with Dunnett’s multiple comparisons test, relative to WT-Lean group for time point. **g**-**h**, Data was analyzed by one-way ANOVA with Tukey’s multiple comparisons test. *p < 0.0332, **p < 0.0021, ***p < 0.0002, and ****p < 0.0001; ns: insignificant. (n = 14 for WT-Lean and WT-HFHS groups; n = 15 for DKI-Lean and DKI-HFHS groups). **i**-**j**, Spatial memory performance (shown in **h**) is inversely correlated with AUC of glucose intolerance test (**e**) in DKI (**j**) but not WT mice (**i**). Data related to Supplementary Figs. 1 and 10. MWM, Morris Water Maze (MWM); AUC, area under the curve

To investigate how diet-induced metabolic dysfunction influences cognitive performance in Alzheimer’s disease (AD) mice, wild-type (WT) and homozygous *App*^*NL−G−F*^*/Mapt*^*hMAPT*^ double knock-in (DKI) mice were exposed to a chronic high-fat, high-sugar (HFHS) diet beginning at 3–4 months of age (Fig. 1a-e). Age-matched littermates maintained on a lean diet served as baseline metabolic controls to distinguish diet-dependent effects from genotype-specific phenotypes. Both WT and DKI mice on the HFHS diet exhibited progressive weight gain compared to lean diet-fed controls (Fig. 1b-c). Within four weeks of diet onset, body weights were significantly different from those of lean-diet mice and continued to rise over 16 weeks, (Fig. 1c). This was accompanied by elevated resting glucose levels (Fig. 1d), consistent with diet-induced insulin resistance [10, 11, 20]. Glucose tolerance testing further confirmed impaired glucose clearance in HFHS-fed mice compared to lean-fed controls (Fig. 1e), with an approximately two-fold increase in blood glucose following an oral glucose bolus.

Cognitive performance in 10-month-old HFHS-fed mice was assessed using the MWM (Fig. 1f-h and Supplementary Fig. S1). During forward memory acquisition training, both WT-HFHS and DKI-HFHS mice had longer swim latencies to locate the hidden platform as compared to lean-fed mice (Fig. 1f). However, by the final training session, prolonged latencies persisted only in DKI-HFHS mice (Fig. 1g). In the forward probe trial, DKI-HFHS mice spent significantly less time in the target quadrant compared to all other groups, indicating impaired memory recall (Fig. 1h). During reversal training, DKI-HFHS mice showed delayed acquisition by the final session (Supplementary Fig. S1a-b), though reversal probe performances did not differ across groups (Supplementary Fig. S1c). Performance in the visible platform task remained consistent across all groups (data not shown), confirming intact visual and motor function.

Correlation analysis between glucose intolerance and probe trial spatial memory performance revealed a significant negative correlation in DKI mice across dietary conditions (Fig. 1j), indicating that increased metabolic impairment is associated with poorer cognitive performance. In contrast, no such relationship was found in WT mice (Fig. 1i). Consistent with these disease-dependent effects, HFHS exposure impairs memory acquisition in both WT and DKI mice, but persistent memory deficits occur only in DKI-HFHS mice. Notably, DKI mice maintained on a lean diet exhibited intact cognitive performance, in contrast to our previous reports for this strain under standard dietary conditions [16, 22]. Overall, these findings indicate a synergistic interaction between metabolic dysfunction and the underlying disease state that impairs cognitive performance.

### HFHS diet alters Trem2, but not classical AD pathology

**Fig. 2:**
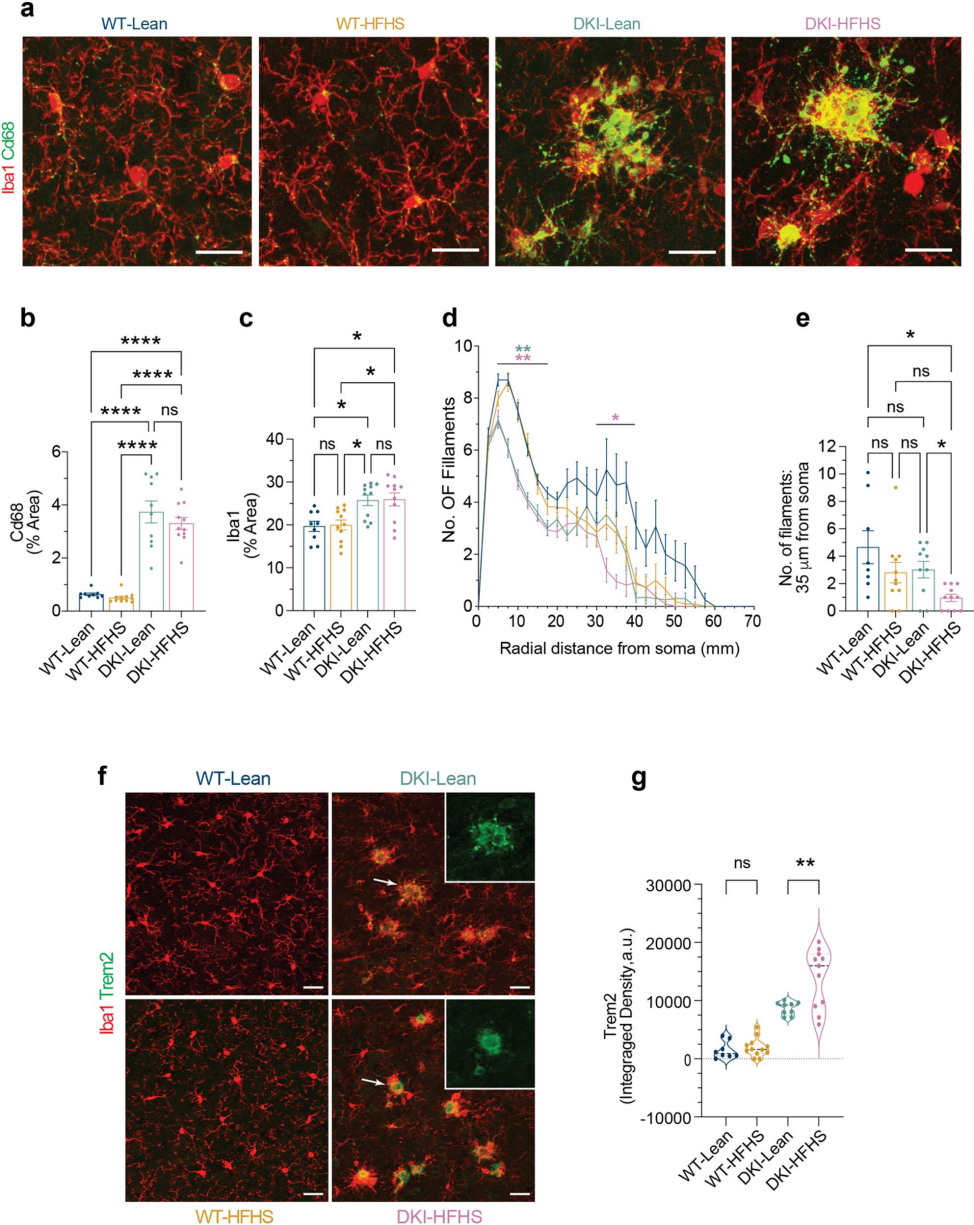
HFHS diet enhances DAM-like morphology and Trem2 proteolytic processing in DKI mice. **a**, Immunofluorescence staining showing the expression levels of Cd68 (green) in microglia morphology (Iba1, red) within the cortical L2-4 region of WT and DKI mice on Lean and HFHS diets. Scale bars: 25 μm. **b-c**, Quantification of Cd68 (**b**) and microglia (**c**) percent coverage areas shown in **a**. (*n = 9* for WT-Lean group; *n* = 11 for WT-HFHS, DKI-Lean and DKI-HFHS groups). **d**, Sholl analysis showing microglia branching complexity across treatment groups. **e**, Quantification of the number of microglia filaments at radial distances 35 μm from the soma. (Date related to Supplementary Fig. 3) **d-e**, Data represents an average of 4–6 cells quantified per mouse, with each point in **e** representing a single mouse. **f**, Representative images of anti-Iba1 (red) and anti-Trem2 (green) immunostaining across treatment groups. Disease-associated Trem2 expression is elevated but exhibits an altered spatial distribution in DKI-HFHS microglia, as shown in the image insets. Scale bar, 25 μm. **g**, Quantification of Trem2 particle integrated signal density as shown in **f**. (**a**,** f**, *n* = 8 for WT-Lean and WT-HFHS groups; *n* = 11 for DKI-Lean and DKI-HFHS groups). All data presented as the mean ± SEM. **b-c**,** e**,**g**, analyzed by two-way ANOVA with Tukey’s multiple comparisons test, *p < 0.0332, **p < 0.0021, and ****p < 0.0001; ns: insignificant

Given previous evidence of glial-mediated synapse loss in DKI mice [22], we explored whether metabolic dysfunction modifies disease-associated microglial (DAM) [29, 30] activation state in 11-month-old mice (Fig. 2). To evaluate whether there was DAM exacerbation in DKI-HFHS mice, we examined co-immunolabeling of Iba1 and Cd68 expression in the mouse cortex (Fig. 2a-c). Cd68 immunoreactivity was elevated to a similar extent in both DKI-Lean and DKI-HFHS mice compared to their WT counterparts (*p* *< 0.0001*, Fig. 2b), indicating greater microglia phagocytic activity associated with Aβ pathology. However, no significant differences were observed between DKI-Lean and DKI-HFHS mice. Iba1⁺ cell density was modestly, but significantly, increased in DKI mice relative to WT controls, independent of diet (*p* < 0.0332; Fig. 2c), consistent with our prior reports of gliosis in this mouse model [16, 22].

Although CD68, a marker of DAM activation [30] did not differ between DKI-Lean and DKI-HFHS mice, and total Iba1 signal was unchanged by diet, we observed subtle alterations in the distal microglial processes of WT-HFHS mice (Fig. 2a). To further evaluate whether the HFHS diet induced microglial morphological remodeling, we performed Sholl analysis on Iba1-labeled cells (Fig. 2d-e; Supplementary Fig. S2a). At 10 μm from the soma, arborization was significantly reduced in both DKI groups compared to WT (*p* *< 0.0001*; Fig. 2d, Supplementary Fig. S2a-b), consistent with disease-associated remodeling. In contrast, at 35 μm, distal microglia arborization was selectively reduced in DKI-HFHS mice (*p* *< 0.0332*; Fig. 2e), with a similar but non-significant trend in WT-HFHS mice, indicating additional diet-specific effects on microglia morphology. While Sholl analysis reveals that HFHS diet consumption specifically diminishes distal branching, other global measures of microglia complexity, such as total process length, branch number, and terminal points, did not differ between DKI-Lean and DKI-HFHS groups (Supplementary Fig. S2c-e). Together, these findings suggest that while disease drives the global retraction of microglial arbors, the HFHS diet further remodels arbor geometry by promoting a more spatially confined branching pattern.

These HFHS-induced morphological changes are consistent with a shift in microglial activation state, potentially modifying or enhancing DAM-related physiology. We therefore examined whether the triggering receptor expressed on myeloid cells 2 (Trem2), a transmembrane receptor explicitly expressed in brain microglia and known AD risk gene that regulates microglial disease activation [31–33], was differentially regulated in DKI-HFHS mice. To visualize Trem2 surface expression, we used an immunolabeling strategy targeting its extracellular domain. As expected, microglia Trem2 expression was upregulated in DKI mice (Fig. 2f, see also Supplementary Fig. S4c-d). Notably, we also observed a diet-induced change in the spatial distribution of Trem2 (Fig. 2f, image insets). In DKI-Lean mice, the Trem2 signal was scattered throughout the periplaque regions, whereas in DKI-HFHS mice, it was more concentrated in compact puncta, resulting in a significant increase in integrated signal intensity (*p* < 0.0021; Fig. 2f, g). The observed remodeling of DAM morphology and redistribution of Trem2 localization indicate a diet-induced modulation of microglia state in the AD brain.

Trem2 has been implicated in the extent of both Aβ and phospho-Tau accumulation [34–36]. Using Imaris-based 3D reconstruction analyses (Supplementary Fig. S3a), we quantified the volume and distribution of Aβ plaques in proximity to microglia to assess potential changes in microglia-plaque interactions. Increased Aβ accumulation occurred in a DKI-dependent manner (Supplementary Fig. S3b). However, volumetric measures of Aβ were indistinguishable between DKI-Lean and DKI-HFHS mice (Supplementary Fig. S3c), and there were no diet-induced changes in microglial plaque association (Supplementary Fig. S3d). We also assessed for HFHS-diet-induced changes in neurite dystrophy and synaptic densities, as soluble Trem2 (sTrem2) has been implicated in these phenotypes as well [35]. Increases in tau phosphorylation markers, AT8 and pThr217, were observed around Aβ-plaques and Trem2 deposits in DKI mice, but with no significant difference between DKI-Lean and HFHS diet-fed mice (Supplementary Fig. S4a-d). We did observe a significant reduction in synaptic densities in DKI-HFHS mice compared to WT-Lean (*p* < 0.0332) and WT-HFHS (*p* < 0.0021) groups, but not relative to DKI-Lean mice (Supplementary Fig. S4e, f). DKI-Lean mice did not exhibit significant synaptic deficits as compared to WT mice on either diet, which aligns with their preserved spatial memory performance (Fig. 1f–h). These findings differ from our prior observations in DKI mice maintained on a standard laboratory diet [16, 22], suggesting that dietary restriction may protect vulnerable synapses from disease pathology. Despite the changes in microglial morphology and synaptic metrics, Aβ deposition and tau phosphorylation were unchanged by HFHS consumption in DKI mice.

### HFHS induces a glial metabolic impairment state in DKI mice

To further investigate glial regulation by metabolic state, we performed single-nucleus RNA sequencing (snRNA-seq) to evaluate group transcriptomic profiles in depth. Pooled cortical and hippocampal tissues from individual mice (*n* *= 4* per group) were processed for nuclei enrichment and isolation. Samples were sequenced, integrated, and batch-corrected for dimensional reduction in UMAP space, followed by Leiden clustering (Supplementary Fig. S5a). Clusters were annotated with major cell type labels based on cluster-specific marker genes, which were identified by differential expression analysis (Supplementary Fig. S5b, Supplementary Table 1). Annotations were cross-validated using the MapMyCells (RRID: SCR_024672) tool from the Allen Brain Cell Atlas (Supplementary Table 2). Post-processing, the dataset yielded a mean sequencing depth of 450 million reads per group, with an average of 30,000 reads and approximately 1,500 unique molecular identifiers (UMIs) per nucleus Supplementary Fig. S5d-f). Several populations of excitatory (ExN) and inhibitory (InN) neurons were identified, along with various glial and non-glial blood-brain barrier cells, with each cell type represented across the groups (Supplementary Fig. S5c). While the proportions of neuronal nuclei remained consistent across conditions, the frequencies of glial populations varied between groups. The number of oligodendrocyte nuclei increased with diet, disease, and their combination, while DKI mice showed elevated microglia (Supplementary Fig. S5g, closed arrows) and reduced astrocyte frequencies, in line with prior findings (Supplementary Fig. S5c) [16, 22]. In UMAP space, a subset of inhibitory neurons appeared exclusively in HFHS-fed mice (Supplemental Fig. 5g, open arrows), indicating a diet-specific response that is examined below. First, we focused on microglia to identify transcriptional changes that may underlie the observed Trem2 alterations, despite the equal number of microglial nuclei in the DKI-Lean and DKI-HFHS groups (Supplementary Fig. S5c).

**Fig. 3:**
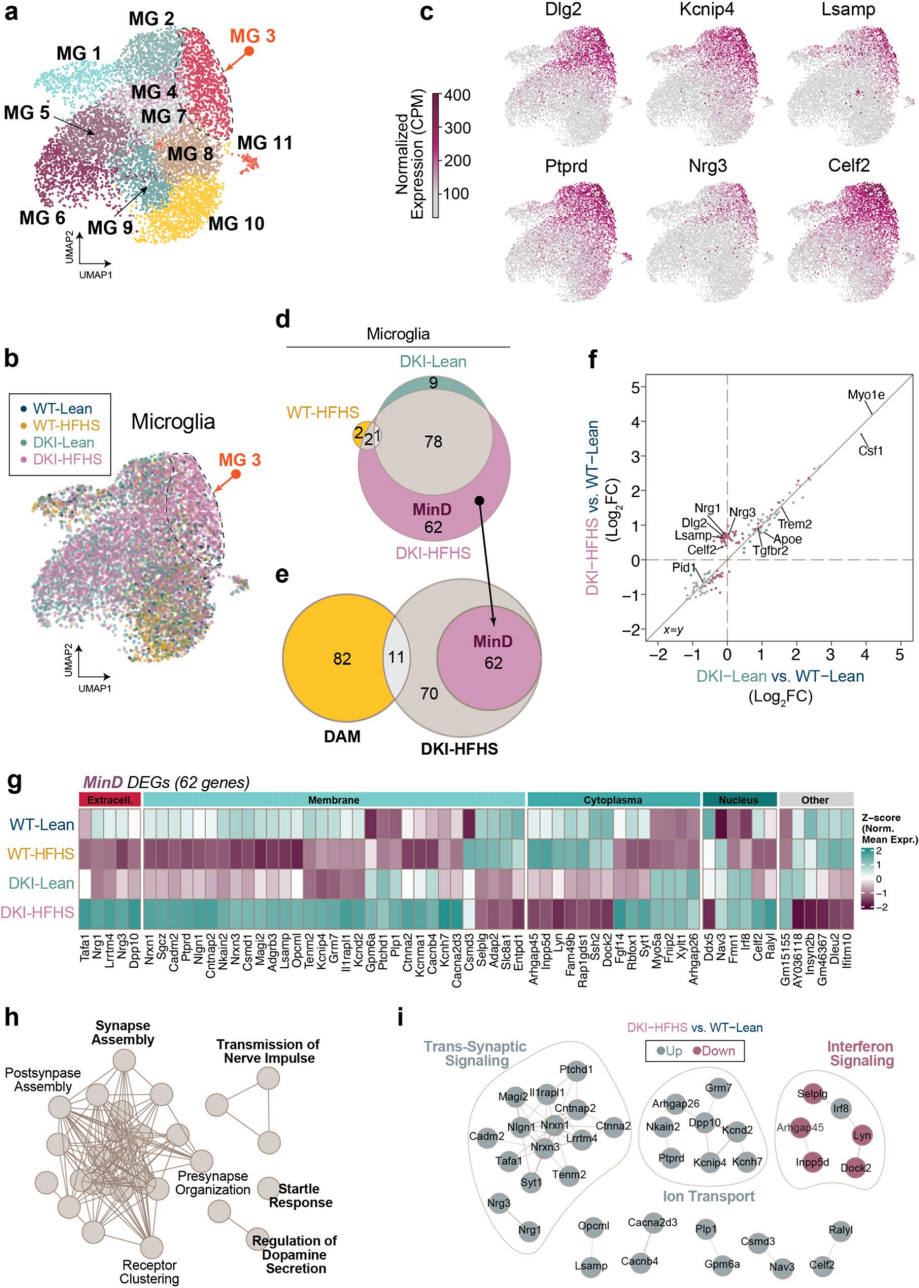
HFHS induces a metabolic impairment transcriptional program atop of DAM gene signature. **a**-**i**, snRNA-seq analysis of microglial nuclei populations isolated from 11-month-old WT and DKI mice under lean or HFHS conditions (n = 4 mice per group). **a**-**b** Subclustering of microglial nuclei shows distinct transcriptomic subsets (**a**). Cluster MG3 (orange arrow) exhibits predominant enrichment of DKI-HFHS microglia. In contrast, other clusters display overlapping contributions from both DKI and WT groups across dietary conditions as shown group nuclei expression profiles in UMAP space (**b**, related to Supplementary Fig. 6). **c**, Nuclei transcript levels of the top six marker genes for the MG3 cluster. **d**, Venn diagram illustrating the overlap of shared DEGs from WT-Lean in WT-HFHS, DKI-Lean, and DKI-HFHS groups. **e**, A Venn diagram showing that genes unique to the DKI-HFHS group do not overlap with published DAM cell state markers, demonstrating a distinct subset of genes are dysregulated due to metabolic impairment in neurodegeneration (MinD). **f**, Correlation plot comparing Log2 fold-changes of DEGs in DKI-Lean and DKI-HFHS microglia relative to WT-Lean microglia. **g**, Cellular localization and group expression patterns of the 62 MinD DEGs identified in **d**-**e**. **h**, Pathway enrichment analysis of 62 DKI-HFHS-specific DEGs depicted in **d**-**f**. **i**, PPI network of genes depicted in **d**-**g** with MCL clustering. Nodes represent individual proteins, while edges represent the interaction between proteins. Color-coded nodes indicate upregulated (green) and downregulated (magenta) genes. Edge thickness represents the relative protein interaction strength. Theme labels represent the main GO Biological Process PPI of the respective subcluster. See Supplemental Table 3 for the complete list of DEGs with corresponding Log2 fold-change values, pathway enrichment, and PPI analysis outputs. Data related to Supplementary Fig. 5–6. UMAP, Uniform Manifold Approximation and Projection; CPM, counts per million; DEGs, differentially expressed genes; PPI, protein-protein interaction; MCL, Markov Cluster algorithm

Microglial nuclei were subset and reclustered for downstream analysis, revealing the selective enrichment of distinct microglial subpopulations across experimental groups (Fig. 3a-c; Supplementary Fig. S6a, b). Leiden clustering yielded 11 microglial subclusters, as shown in UMAP space (Fig. 3a). Clusters MG8, MG9, MG10, and MG11 predominantly consisted of homeostatic microglia, while clusters MG4, MG5, MG6, and MG7 were enriched in DKI mice, expressing marker genes consistent with previously described DAM profiles (Supplementary Fig. S6b, c). Notably, cluster MG3 was selectively enriched in DKI-HFHS mice (arrows in Fig. 3a, b; Supplementary Fig. 6b), representing a unique transcriptional state associated with metabolic stress in the context of neurodegeneration, which we refer to here as a *metabolic* *impairment in* *neurodegeneration* (MinD) state. Differential gene expression (DEG) analysis (complete list in Supplementary Table 3) identified *Dlg2*, *Kcnip4*, *Lsamp*, *Ptprd*, *Nrg3*, and *Celf2* as the top marker genes selectively enriched and upregulated in MG3 relative to all other microglial clusters (Fig. 3a, c).

To contextualize the MinD transcriptional program, we performed group-wise enrichment analyses across the total microglial population, comparing each experimental group to WT-Lean. This pan-microglial approach minimized potential biases arising from differences in nuclei counts within subcluster MG3. We performed DEG analysis applying a significance threshold of adjusted *p* < 0.05 and a log_2_ fold-change cutoff of >|0.25|. A Venn diagram of significant DEGs (Fig. 3d, Supplementary Table 3) revealed 78 genes shared between DKI-HFHS and DKI-Lean groups, while 62 MinD genes were uniquely altered in DKI-HFHS microglia. Next, we sought to determine whether the MinD transcriptional program overlaps with DAM-associated signatures or is distinct. Using curated microglia cell-state gene sets [30], we assessed the relative expression levels of DAM and other disease-associated microglia cell-state genes (Supplemental Fig. 6c-d). Canonical microglial signatures were stratified between WT and DKI groups, consistent with disease-associated activation (Supplementary Fig. S6d). In contrast, MinD genes form a subset of DKI-HFHS DEGs with minimal overlap with canonical microglial state signatures, as shown for DAM (Fig. 3e) and other states (Supplementary Fig. S6e), indicating a distinct metabolic impairment transcriptional program in the AD mice. To visualize the correlation of gene expression between the two DKI groups, we plotted the log_2_ fold-changes for DKI-HFHS and DKI-Lean microglia relative to WT-Lean (Fig. 3f). This revealed a strong positive correlation in disease-associated gene expression, with overlapping (gray), DKI-Lean-specific (green), and DKI-HFHS-specific (magenta, MinD) genes distributed along the identity line. Notably, a distinct cluster of points (magenta), representing nearly half of the DKI-HFHS-specific DEGs, deviated from the linear trend, further confirming that these MinD DEGs define a transcriptional state driven by the compound effects of diet and disease.

To better understand the biological context of genes specifically altered in DKI-HFHS microglia, we organized the 62 DEGs in a heatmap according to their predicted cellular localization and visualized their relative expression levels across each group (Fig. 3g). Of the specific DKI-HFHS genes clustered in the correlation plot (Fig. 3f), a substantial proportion encoded plasma membrane-associated or secreted proteins (Fig. 3g). Pathway enrichment analysis (Fig. 3h, Supplementary Table 3) highlighted gene sets involved in synaptic assembly, presynaptic organization, and synaptic receptor clustering. A protein-protein interaction network analysis (Fig. 3i, Supplementary Table 3) identified dense connectivity among upregulated genes involved in trans-synaptic signaling and ion transport. In contrast, a subset of genes associated with the interferon signaling pathway was downregulated, except for Irf8, a transcription factor implicated in microglial immune activation [37, 38].

**Fig. 4:**
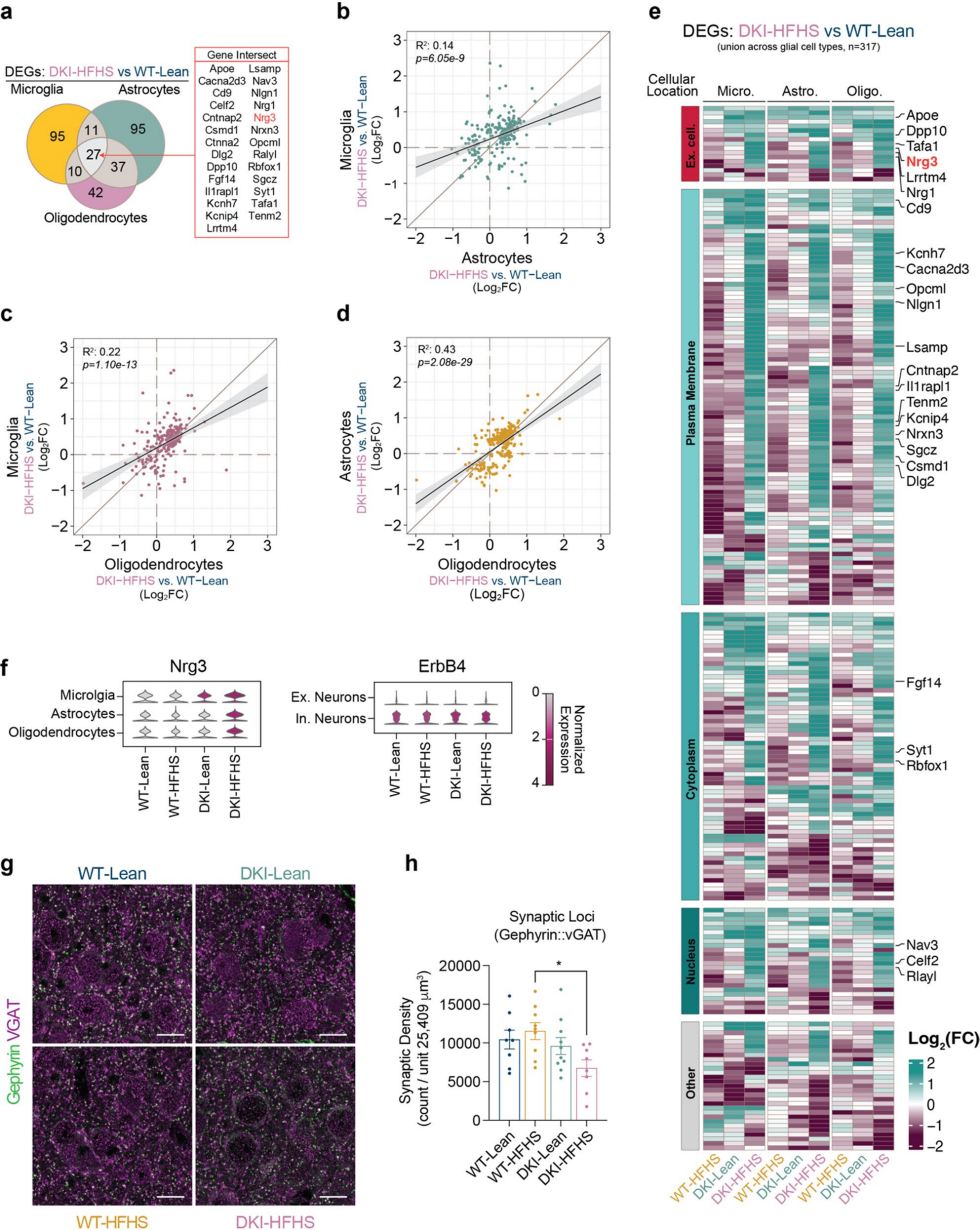
Loss of inhibitory synapses in DKI-HFHS mice corresponds with pan-glial MinD state. **a**-**e**, Shared DEGs across microglia, astrocytes, and oligodendrocytes show positive intercellular correlations and reflect a shared MinD state transcriptional program upregulated in DKI-HFHS mice. **a**, Venn diagram of shared and unique DEGs across microglia, astrocytes, and oligodendrocytes in the DKI-HFHS group, with the 27 DEGs common to all glial types listed. **b**-**d**, Pairwise correlation plots comparing Log2 fold-changes of shared DEGs from **a** for microglia vs. astrocytes (**b**), microglia vs. oligodendrocytes (**c**), and astrocytes vs. oligodendrocytes (**d**), for the DKI-HFHS vs. WT-Lean comparison, each showing a positive correlation. **e**, Union of DEGs identified in (**a**-**d**) organized by protein cellular location, highlighting a glial-specific transcriptional program linked to metabolic impairment in DKI-HFHS mice. Data Related to Fig. 3 and Supplementary Fig. 6–8. See Supplemental Tables 3 and 4 for the complete DEG list. **f**, Stacked-violin plots showing normalized transcriptomic levels of Nrg3 in glial and ErBB4 in neuronal nuclei populations. **g**, **h**, Representative immunostaining (**g**) and corresponding colocalization quantification of Gephyrin with vGAT-positive synapses (**h**). Imaging analysis was performed within cortical L2/3. (n = 9 for WT-Lean group; n = 11 for WT-HFHS, DKI-Lean, and DKI-HFHS groups.) For (**h**) data presented as the mean ± SEM and analyzed by two-way ANOVA with Tukey’s multiple comparisons test, *p < 0.0332. MinD, metabolic impairment in neurodegeneration; DEG, differentially expressed genes; micro, microglia; astro, astrocytes; oligo, oligodendrocytes

Pan-glial transcriptomic remodeling was evident in the DKI-HFHS mice, extending beyond microglia to astrocytes and oligodendrocytes (Fig. 4a-e, Supplementary Fig. S7 and S8). Significantly greater GFAP-positive astrocyte signal was detected in the cerebral cortex of both DKI groups (Supplementary Fig. S7a, b), coinciding with significant disease-associated activation compared to WT controls (*p <* 0.0001). However, astrocytes (Supplementary Fig. S7c) and oligodendrocytes (Supplementary Fig. S8a, b) in DKI-HFHS mice displayed transcriptomic profiles with properties resembling the microglial MinD state (Fig. 3, Fig. S6a-c). In UMAP space, distinct sets of genes were uniquely enriched from DKI-HFHS mice but not DKI-Lean, defining MinD transcriptional programs in both astrocytes and oligodendrocytes (Supplementary Fig. S7c, f and S8a-b, e). Differential expression analysis confirmed that DKI-HFHS mice exhibited the most significant number of transcriptional alterations within both astrocytes and oligodendrocytes (Supplementary Fig. S7d and 8c). Similar to the comparison of microglial MinD genes with the DAM profile, the astrocytic MinD genes form a subset of DKI-HFHS DEGs having minimal overlap with published DAA-associated genes (Supplementary Fig. S7e) [39]. In astrocytes and oligodendrocytes, log_2_ fold-change comparisons relative to WT-Lean revealed discrete separation between DKI-Lean and DKI-HFHS groups (Supplementary Fig. S7g and S8d). Although many changes of the DKI-HHS mice were unique to individual glial populations, 27 MinD DEGs were shared across all three glial types (Fig. 4a). Correlation analyses demonstrated strong positive associations in DEG expression between microglia, astrocytes, and oligodendrocytes (Fig. 4b–d). The union of glial diet-induced DEGs in DKI-HFHS mice, organized by predicted cellular localization (Fig. 4e), define a shared MinD transcriptional program in response to combined metabolic and neurodegenerative stress.

Among the MinD state genes selectively upregulated in DKI-HFHS microglia, neuregulin 3 (Nrg3) consistently emerges as a gene of interest (Fig. 3c, f, g, and i). Nrg3, along with the upregulated Nrg1, belongs to the neuregulin family of secreted ligands known to modulate synaptic function through ErbB receptors [40]. Nrg3 transcripts were also upregulated in astrocytes and in oligodendrocytes of the DKI-HFHS mice (Fig. 4a, f,e; Supplementary Fig. S7f and Supplementary Fig. S8e). While Nrg1 can signal through ErbB2, ErbB3, and ErbB4 [40–42], Nrg3 primarily binds to ErbB4, a receptor selectively enriched at inhibitory synapses (Fig. 4f) [40, 42–44]. Therefore, we assessed inhibitory synapse density using gephyrin–vGAT co-localization and observed reduced inhibitory synapse loci in DKI-HFHS mice relative to WT-HFHS controls (*p <* 0.0332), whereas no significant differences were detected between lean-diet groups (Fig. 4g, h, and Supplementary Fig. S4e, f). These data define a molecular pathway specifically engaged by the combination of AD pathology and diet-induced insulin resistance, leading to altered neuroglial interactions.

### HFHS triggers metabolic impairment program in Meis2 + L2 neurons

**Fig. 5:**
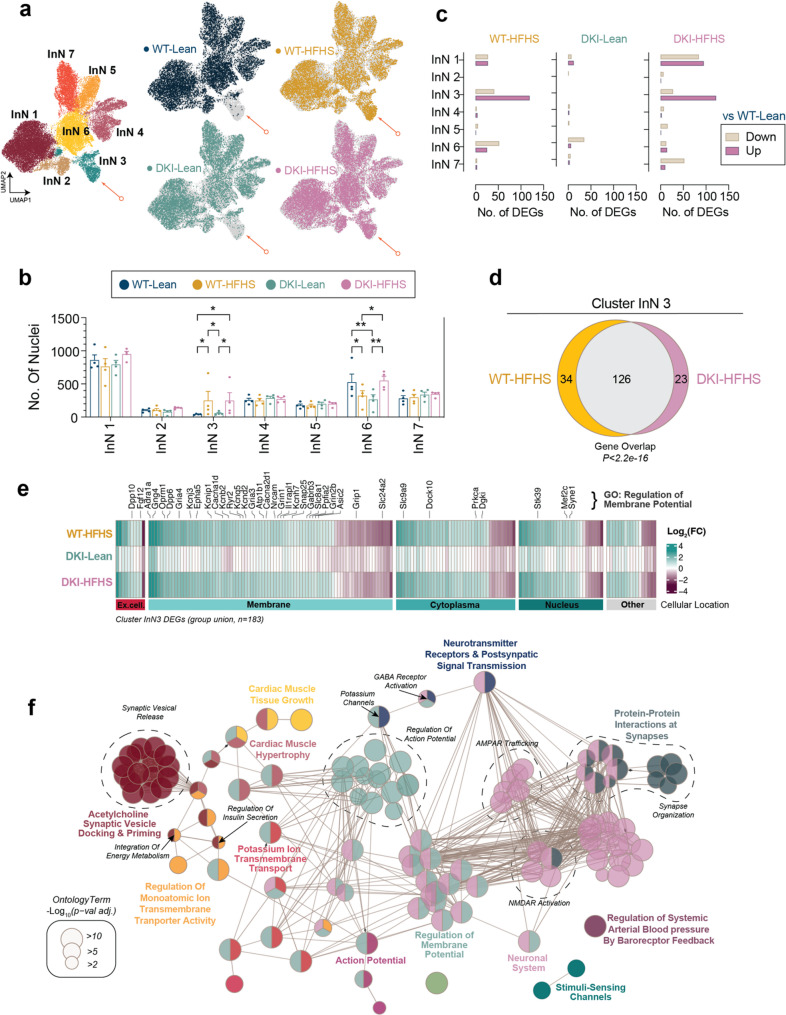
HFHS diet induces a metabolic stress transcriptional program in specific inhibitory neurons. **a**, UMAPs showing subclustering of InN nuclei (left) and group expression profiles (right). Orange arrows point to subcluster InN 3, which exhibits selective nuclei enrichment in HFHS diet groups. **b**, Mean nuclei frequencies of subclusters shown in a. Data are presented as mean ± SEM and analyzed by two-way ANOVA test with Holm-Šidák multiple comparisons correction. *p < 0.0332, **p < 0.0021. **c**, DEG counts for InN subclusters of each respective group vs. WT-Lean. **d**, Venn diagram illustrating the overlap of shared DEGs between WT-HFHS and DKI-HFHS groups for cluster InN3 shown in c. Significance P-value for gene overlap was computed using Fisher’s Exact Test. **e**, Heatmap of the Log2 fold-changes of the union of all DEGs depicted in **d**, organized by each gene’s protein cellular location. Highlighted Log2 fold-changes are of all the genes represented by the ‘Regulation of Membrane Potential’ node shown in **f**. **f**, Pathway enrichment analysis of nuclei subcluster InN 3 using the union of DEGs depicted in **d**. The Enrichment Map represents clustered nodes of GO Biological Process pathways, with edges connecting pathways shared by many genes. Theme labels represent the primary GO term associated with each node subcluster, with colors coordinated to enhance visualization. Node sizes correspond to the -Log2 transformed adjusted p-value for each GO term gene set enrichment. See Supplemental Table 5 for a complete list of DEGs and pathway enrichment analysis outputs. UMAP, Uniform Manifold Approximation and Projection; InN, inhibitory neurons; vs., versus. GO, gene ontology

**Fig. 6:**
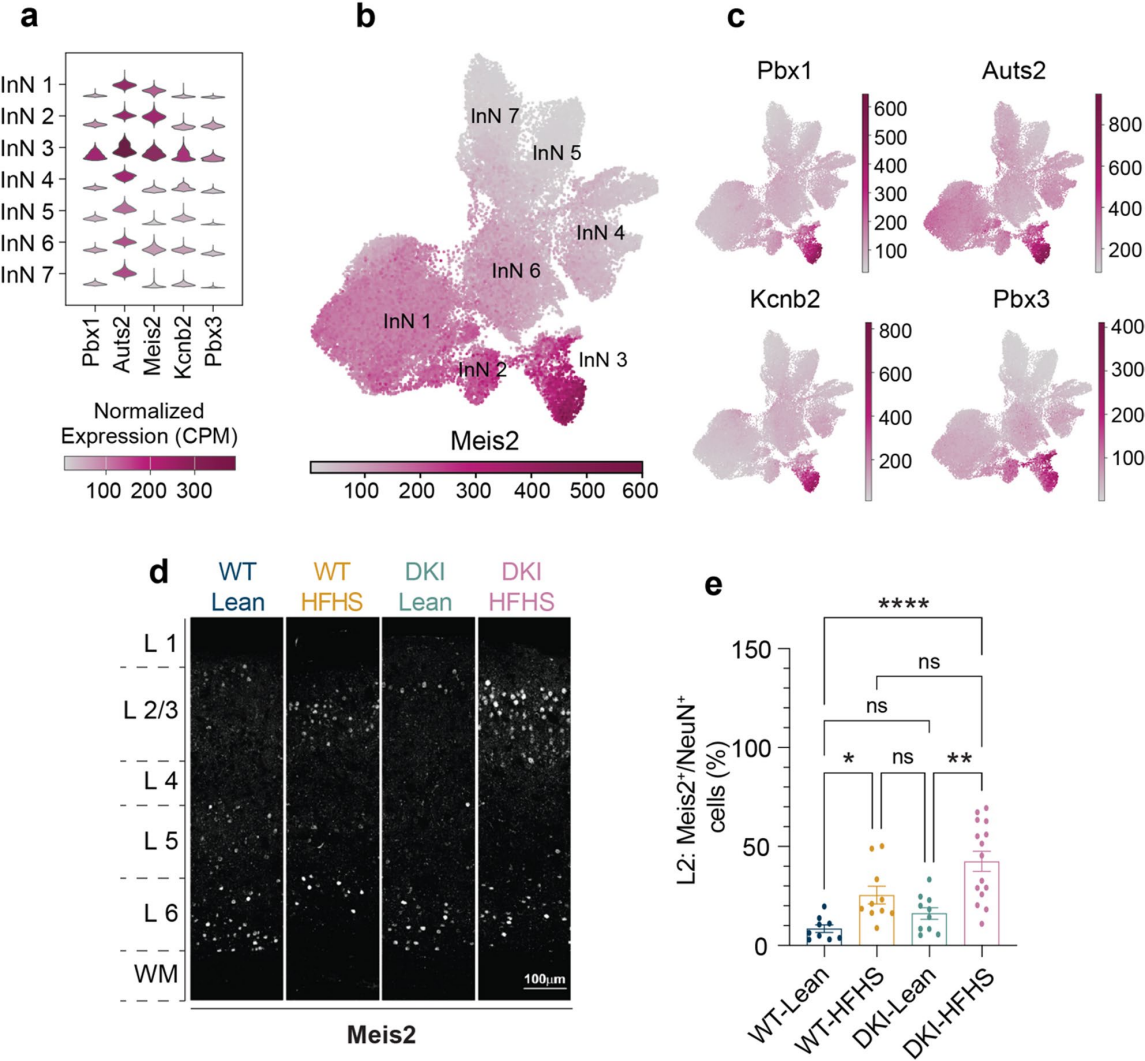
HFHS diet induces Meis2 upregulation in L2 inhibitory neurons with amplified levels in DKI mice. **a**-**c**, Top marker genes for subcluster InN 3, identified as cortical L2 Meis2+ InNs. See Supplemental Table 5 for the full list of cluster marker genes. Data related to Supplemental Fig. 9. **a**, Stacked-violin plot depicting normalized transcript levels of the top five InN 3 cluster marker genes, displayed across all InN subclusters. **b**, **c** The transcript levels of the top genes shown in a, displayed in UMAP space. Scale bars, normalized CPM. **d**, Representative images demonstrating Meis2 expression levels across cortical layers. There is a diet-induced increase of Meis2 within cortical L2, which is further increased in DKI-HFHS mice. **e**, Quantification of the percentage of Meis2+ cells as a proportion of the total number of NeuN+ cells located in cortical L2 as represented in **d**. (n = 9 for WT-Lean group; n = 11 for WT-HFHS, DKI-Lean, and DKI-HFHS groups.) Data presented as the mean ± SEM and analyzed by two-way ANOVA with Tukey’s multiple comparisons test, *p < 0.0332, **p < 0.0021, and ****p < 0.0001; ns: insignificant. CPM, counts per million; InN, inhibitory neurons; L2, layer 2; WM, white matter

We considered whether neuronal gene expression varied in parallel with these glial changes. Subclustering of inhibitory neurons revealed seven transcriptionally distinct groups, with a selective enrichment of nuclei from HFHS-fed mice in cluster InN3 (arrows, Fig. 5a, b). DEG analysis showed a high number of transcriptional changes in InN3 nuclei in both WT-HFHS and DKI-HFHS mice, but not in DKI-Lean mice, compared to WT-Lean (Fig. 5c). The majority of DEGs were shared between WT-HFHS and DKI-HFHS (Fig. 5d), indicating a common transcriptional response to HFHS exposure (Fig. 5e). Pathway enrichment analysis of the combined DEGs revealed densely interconnected nodes associated with synaptic organization, vesicle release, and trans-synaptic signaling, with the most prominent gene ontology terms linked to the regulation of membrane potential (Fig. 5f, Supplementary Table 5). These signatures point to altered cellular excitability as a defining feature of InN3 neurons under metabolic stress. Cell type classification identified InN3 nuclei as Meis2⁺ Layer (L) 2/3 inhibitory neurons (Fig. 6a-b, d-e, and Supplementary Table 1). Meis2, like the other top five marker genes, *Pbx1*,* Auts2*,* Kcnip2*, and *Pbx3*, has strongly elevated transcript levels in InN3 nuclei. In vivo, Meis2 expression was also elevated in L2/3 cortical neurons in response to HFHS diet, with the most substantial increase observed in DKI-HFHS mice (Fig. d-e). As a transcription factor linked to pancreatic glucose dysregulation [45, 46], increased Meis2 expression suggests a parallel metabolic impairment program in the brain, with cortical L2/3 Meis2⁺ inhibitory neurons exhibiting selectively sensitivity to HFHS-induced metabolic stress.

Recently, reports indicated that the HFHS diet promotes insulin resistance by thickening perineuronal nets (PNNs) around hypothalamic AgRP neurons, thereby limiting their response to peripheral signals, such as insulin [47]. Since PNNs are also known to ensheathe Pvalb⁺ interneurons in the cortex [48, 49], we investigated whether HFHS-associated changes in Meis2⁺ inhibitory neurons align with cortical PNN remodeling. Utilizing Wisteria floribunda agglutinin (WFA) labeling combined with Pvalb immunostaining, we found that PNNs were predominantly localized to cortical L4 (Supplementary Fig. S9 a). A trend toward increased PNN density was observed in response to both diet and disease (Supplementary Fig. S10c); however, this increase was restricted to deeper cortical layers, with no PNN expansion detected in L2/3 (Supplementary Fig. S9a). Notably, Meis2⁺ neurons in L2/3 did not co-localize with Pvalb and Pvalb⁺ cell numbers remained unchanged across groups (Supplementary Fig. S9b-d). These findings suggest that the transcriptional changes observed in Meis2⁺ neurons are unlikely to result from PNN-associated insulin resistance, distinguishing them from mechanisms described in hypothalamic AgRP neurons [47].

### ExNs have distinct yet convergent transcriptomics patterns of synaptic dysregulation

**Fig. 7:**
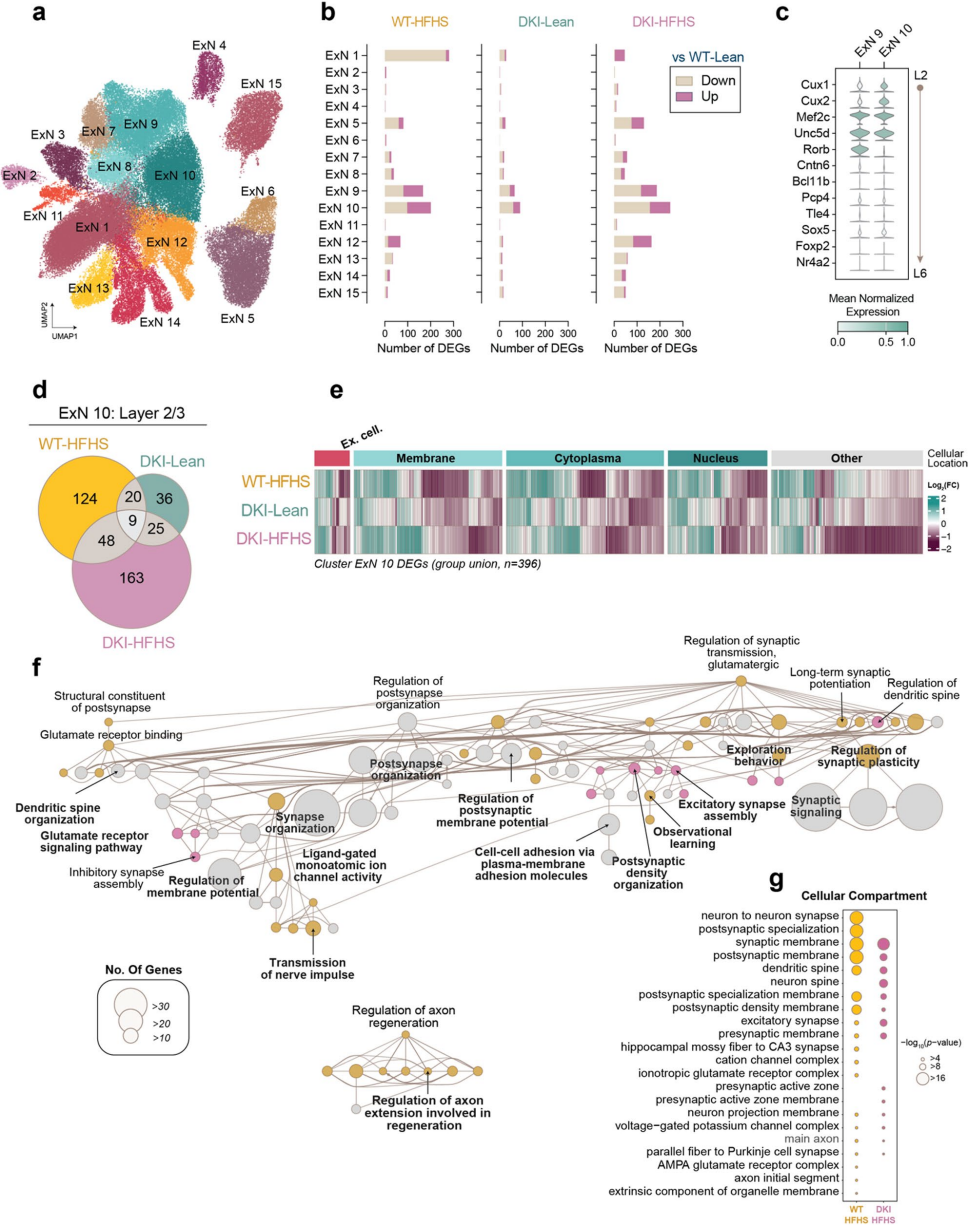
Layer 2–4 ExNs show distinct disease- and diet-specific transcriptional changes with convergent synaptic pathway dysregulation. **a**, UMAP of subclustered ExN nuclei populations. **b**, DEGs counts per ExN subcluster for each group compared to WT-Lean. ExN 9 and ExN 10 nuclei subpopulations exhibit genetic dysregulation in response to disease, diet, and the combination of the two. (See Supplemental Table 6 for full DEG list). **c**, Stacked-violin plots showing cortical layer markers corresponding to L2-L6. Marker genes for L2 and L3 are enriched in nuclei cluster ExN 10, while L3 and L4 marker genes are enriched in nuclei cluster ExN 9. **d**, Venn diagram showing DEGs overlap between groups as compared to WT-Lean mice for nuclei cluster ExN 10 (shown in **a**-**c**). **e**, Heatmap of the Log2 fold-changes of the union of all cluster ExN 10 DEGs depicted in **b**, **d**, organized by each gene’s protein cellular location. **f**, Merged pathway enrichment map for WT-HFHS and DKI-HFHS dysregulated genes, organized using a tree layout where nodes are arranged hierarchically to emphasize parent-child relationships between pathways. Nodes are colored by gene set contribution (yellow, WT-HFHS; magenta, DKI-HFHS; grey, equal contributions), and node size reflects the number of genes. **g**, Dot plot of GO Cellular Component terms resulting from the enrichment analysis for ExN 10 cluster DEGs for WT-HFHS and DKI-HFHS nuclei samples. Dot size indicates the P-value of significance of each term. ExN, excitatory neurons; DEGs, differentially expressed genes; L, layers

We next investigated whether excitatory neurons exhibit a transcriptomic signature of metabolic impairment in response to HFHS. Fifteen excitatory subclusters were identified (Fig. 7a), with ExN9 and ExN10 showing the highest number of DEGs following HFHS exposure (Fig. 7b). A smaller set of disease-associated DEGs was also detected in DKI-Lean neuronal nuclei. Cell type classification (Supplementary Table 1) identified these clusters as L2-L4 excitatory neurons, with ExN9 enriched for L3/4 markers and ExN10 for L2/3 markers (Fig. 7c). Given the sensitivity to metabolic impairments observed in Meis2⁺ L2 inhibitory neurons, we focused our analysis on cluster ExN10, representing L2/3 excitatory neurons. DEG analysis revealed distinct, group-specific gene sets with minimal overlap (Fig. 7d, Supplementary Table 6). However, the resulting expression signatures were strikingly similar across conditions, indicating transcriptional convergence despite divergent gene identities (Fig. 7e). Merged pathway analysis with hierarchical clustering confirmed this pattern. While some functional nodes were specific to WT-HFHS (yellow) or DKI-HFHS (magenta) DEGs, the majority reflected shared contributions (gray) (Fig. 7f, see Supplementary Table 6 for all pathway enrichment outputs). Like the pathway enrichment analysis of Meis2⁺ L2 inhibitory neurons (Fig. 5f), the most highly connected pathways were associated with synapse organization and trans-synaptic signaling, driven by genes localized to synaptic membranes (Fig. 7f, g). Additionally, a node cluster of separate axon-specific pathways was uniquely enriched in WT-HFHS (Fig. 7f). These findings suggest that excitatory neurons exhibit a transcriptomic signature of metabolic impairment that converges on altered synaptic function through partially overlapping yet mechanistically distinct programs.

## Discussion

This study identifies glial and L2 inhibitory neuronal transcriptional responses to diet-induced insulin resistance in a knock-in mouse model of AD. Chronic HFHS exposure of DKI mice was associated with impaired spatial learning, the emergence of a shared glial metabolic impairment program (MinD), and selective upregulation of Meis2 in cortical Layer 2 inhibitory neurons. These cellular responses occurred without increased Aβ or tau accumulation, indicating that metabolic stress alters brain cell states independently of overt changes in classical AD pathology. The approaches described in this study provide a basis to understand the capacity of brain insulin resistance to exacerbate AD-related neurological dysfunction. The use of double-knock-in AD mouse models, chronic diet-induced metabolic syndrome, and single-nucleus transcriptomic profiling of brain tissue strengthens the translational relevance of our observations. Together, these data underscore the critical role of metabolic dysfunction in altering age-dependent AD pathophysiology.

### Metabolic dysfunction as a disease modifier of Alzheimer’s progression

Metabolic dysfunction is increasingly recognized as a critical modifier of AD risk and progression [1, 2, 5, 50–52] yet the underlying mechanisms have been ill-defined. In our mouse model, chronic HFHS exposure induced obesity, hyperglycemia, and glucose intolerance in WT and DKI mice. However, spatial memory impairment was only observed in DKI-HFHS mice. DKI mice maintained on a lean diet performed comparably to WT controls, despite no difference in Aβ and tau pathology between DKI lean and HFHS mice. Differences in body weight and glucose tolerance across dietary conditions (Supplementary Fig. S10, Fig. 1c-e) indicate that systemic metabolic effects of chronic HFHS exposure do not alone account for the observed behavioral phenotypes in WT and DKI mice. This reinforces the concept that brain insulin resistance contributes to AD-associated cognitive decline [5, 53, 54]. The results also highlight that dietary composition plays a crucial role in mitigating cognitive deficits associated with AD [18, 55–57], independent of the level of protein aggregate pathology.

### Glial metabolic stress responses and microglia-synapse interactions

Recent studies have expanded beyond M1/M2 classifications to recognize a spectrum of microglial activation states associated with neurodegenerative diseases [30, 58, 59]. Using snRNA-seq, we identified a unique glial transcriptional program selectively induced in DKI-HFHS mice that we term the *metabolic* *impairment in* *neurodegeneration* (MinD) state. This state was most prominent in a microglial subcluster, MG3, defined by the selective upregulation of genes involved in synaptic regulation (e.g., Dlg2, Kcnip4, Lsamp, Ptprd, and Nrg3), transmembrane signaling, and Aβ pathology. Notably, the MinD signature is distinct from known DAM and inflammatory microglia profiles (Fig. 3e, Supplemental Fig. 6e) [30]. Furthermore, it is absent in WT-HFHS mice, indicating that it represents a specific response arising from the intersection of diet-induced metabolic stress and AD pathology.

 In vivo, we observed altered Trem2 expression and processing in DKI-HFHS microglia. Transcriptomic analysis revealed several MinD-upregulated genes, including Celf2 and Irf8, alongside downregulation of Inpp5d, all of which are known regulators of Trem2 signaling and processing. Celf2, an RNA-binding protein, regulates alternative splicing of Trem2 to produce soluble Trem2 (sTrem2), though its effects on sTrem2 abundance remain unclear [60, 61]. Irf8, a transcription factor essential for microglial identity and activation, has been shown to regulate Trem2-dependent transcriptional programs and disease-associated microglia states [37, 38]. Conversely, Inpp5d, a negative regulator of PI3K-AKT signaling downstream of Trem2, was downregulated, suggesting a shift toward enhanced Trem2 signaling under metabolic stress [62]. Because sTrem2 can bind Aβ and influence its clearance or aggregation [35], these transcriptomic changes may contribute to the altered Trem2 phenotype observed in vivo. This altered expression profile may, in turn, link diet-induced transcriptional shifts to alterations in Aβ dynamics under metabolic stress.

Among microglial MinD genes, Nrg3 emerged as a candidate linking glial transcriptional shifts to inhibitory synaptic signaling. Nrg3 is a selective ligand for ErbB4, an inhibitory synapse-enriched receptor [43, 63, 64]. ErbB4 signaling itself has been linked to metabolic dysregulation and clinical obesity [65]. In the DKI-HFHS cortex, Nrg3 transcript levels were increased, suggesting enhanced microglia-derived Nrg3-ErbB4 engagement. Indeed, inhibitory synapse density was substantially reduced. We also observed Nrg3 upregulation in astrocytes and oligodendrocytes, indicating that multiple glial populations contribute to ErbB4-mediated signaling. We hypothesize that the MinD transcriptome in glia alters inhibitory circuit integrity at least in part through Nrg3-mediated mechanisms.

While our in vivo validation of MinD focused on microglia, the astrocytes and oligodendrocytes of DKI-HFHS mice also exhibited strikingly similar MinD-like transcriptional changes, which are distinct from canonical disease-associated activation states. Similar to microglia, astrocytic MinD genes are distinct from DAA-associated transcriptional programs. Previously, we reported that the transcriptional responses of astrocytes and oligodendrocytes follow delayed inflammatory trajectories compared to microglia in the DKI strain, becoming more pronounced at 20 months than at 10 months [16]. The emergence of a robust glial-wide MinD state at 11 months suggests an earlier and coordinated adaptation to metabolic stress across glial populations. Therefore, a glia-wide MinD state may represent an early step in the pathological cascade linking metabolic dysfunction to AD progression. Surprisingly, several shared glial MinD state genes (e.g., Lrrtm4, Dlg2, and Ptprd) are risk factors for autism spectrum disorders [66, 67], another condition associated with metabolic dysfunction [68]. This highlights the potential broader significance of the glial MinD states in other metabolic disorders.

### Meis2 upregulation in inhibitory neurons under metabolic stress

One of the most striking findings from our study was the selective upregulation of the transcription factor Meis2 in L2/3 inhibitory neurons. In accordance with our transcriptomic analysis, in vivo Meis2 protein levels were elevated in HFHS-fed mice, with further increased expression in DKI-HFHS mice. This robust diet-specific response was absent in DKI-Lean mice, an indication that Meis2 acts as a metabolic stress–responsive factor that is further dysregulated in the AD context. Meis2 is known to form transcriptional complexes with Pbx1 and Pbx3 in pancreatic β-cells where it participates in glucose-responsive gene regulation [69–72]. Remarkably, these same genes are among the top five cell identity markers in Meis2^+^ L2 inhibitory neurons. These findings suggest that Meis2⁺ neurons may engage a metabolic homeostasis program in response to dietary stress, paralleling its transcriptional role in pancreatic β-cells as a mitigator of hyperglycemic signals. Other top genes enriched in this cell population include Auts2, an autism risk gene [73], and Kcnb2, which regulates both insulin secretion [74] and neuronal excitability [75, 76].

Meis2^+^ inhibitory neurons exhibited transcriptomic enrichment for pathways regulating vesicle release, synaptic organization, and membrane potential, which are selectively altered under HFHS-diet conditions. Notably, these activated pathways were identical in both WT-HFHS and DKI-HFHS mice, indicating that this transcriptional response is broadly induced by metabolic stress. However, only DKI-HFHS mice exhibited cognitive impairments, suggesting that Meis2 activation alone is insufficient to drive dysfunction and may represent an adaptive response under pathophysiological conditions. In parallel, adjacent L2-4 excitatory neurons exhibit distinct transcriptional changes in response to disease and diet, but converge on similar signaling cascades, all of which are linked to synaptic signaling. These coordinated transcriptional changes across L2-4 inhibitory and excitatory neurons point to focal remodeling of upper-layer cortical circuitry under metabolic stress.

Whether these transcriptional changes are compensatory and limiting dysfunction versus being pathogenic and driving circuit destabilization remains unclear. Collectively, the observed transcriptomic profile of Meis2 + inhibitory neurons suggests potential alterations in cellular excitability in response to diet-induced metabolic stress. This study did not directly assess neuronal firing properties or synaptic potentiation of these neurons. Future studies combining electrophysiological recordings with conditional genetic manipulation of Meis2 will be necessary to determine whether Meis2 regulates excitability in inhibitory neurons. In particular, directly comparing Meis2⁺ neuron responses between WT-HFHS and DKI-HFHS conditions would help determine whether metabolic stress alone is sufficient to alter neuronal function or whether these changes emerge specifically in the disease context. In addition, defining the transcriptional targets of Meis2 in this setting will be important to understand how metabolic stress engages a regulatory program.

### Transcriptomic consequences of insulin signaling and resistance

Metabolic dysfunction, with or without AD, has been associated with impaired brain insulin signaling, including disruptions in IRS-1 and AKT pathways, indicative of a brain insulin-resistant state [4–6, 54, 77]. These disruptions have motivated clinical trials of central insulin delivery as a therapeutic strategy for AD [8, 78]. In contrast, our study focused on the transcriptional consequences of insulin resistance. We observed pronounced gene expression changes across glial types and within a specific class of inhibitory neurons in response to metabolic stress. Future work will determine whether these transcriptional shifts are driven by canonical insulin signaling pathways or mediated by alternative mechanisms that link metabolic dysfunction to gene regulatory networks. Reduced expression of the anti-aging gene Sirtuin 1 has been suggested to play a role in age-related synaptic loss and glial expression changes [79–81]. However, neither transcriptomic nor immunohistological assessment revealed altered Sirtuin 1 mRNA or protein in the HFHS-fed DKI model (L. Nicholson and S. Strittmatter, unpublished observations).

## Conclusion

Using a diet-induced model of systemic insulin resistance, we demonstrate that metabolic stress selectively alters brain cellular states in AD independently of overt changes in classical protein aggregate pathology. Chronic HFHS exposure in AD knock-in mice was associated with the emergence of a shared glial metabolic impairment program and transcriptional remodeling of Meis2⁺ inhibitory neurons. These findings indicate that metabolic dysfunction can influence cortical circuit integrity through coordinated glial and neuronal responses. Clinically, metabolic dysfunction has been associated with worsened outcomes in early-stage AD and paradoxical protection later in disease progression [82, 83], suggesting a dynamic interplay between metabolic and neurodegenerative processes. Our results also suggest that metabolic stress modifies cellular responses in the AD brain in ways not captured by conventional neuropathology. Future studies will investigate whether the glial MinD state represents a neuroprotective adaptive response, a maladaptive driver of pathology, or a context-dependent state that shifts over the course of disease progression. By modeling the compound effects of diet and disease, this study provides a tractable framework for understanding how metabolic dysfunction influences neurodegenerative processes and highlights candidate cellular pathways linking metabolic stress to cortical circuit dysfunction in AD.

## Supplementary Information

Below is the link to the electronic supplementary material.

Supplementary Figures

Supplementary Table 1

Supplementary Table 2

Supplementary Table 3

Supplementary Table 4

Supplementary Table 5

Supplementary Table 6

## Data Availability

All data generated and/or analyzed during this study are included in this published article and its supplementary information files. The transcriptomics dataset supporting the conclusions of this article is available in the NCBI’s Gene Expression Omnibus (GEO) repository, GSE262426 at [https://www.ncbi.nlm.nih.gov/geo/query/acc.cgi?acc=GSE262426].

## Abbreviations

AD: Alzheimer’s disease
Aβ: Amyloid-β
APP: Amyloid precursor protein
DAM: Disease-associated microglia
DKI: Double knock-in (*App*^*NL-G-F*^*/Mapt*^*hMAPT*^) mice
DEG: Differentially expressed gene
ExN: Excitatory neuron
GFAP: Glial fibrillary acidic protein
HFHS: High-fat, high-sugar diet
InN: Inhibitory neuron
Iba1: Ionized calcium binding adaptor molecule 1
L2-4: Layer 2-4
Mapt, tau: Microtubule-associated protein, tau
Meis2: Meis homeobox 2
MinD: Metabolic impairment in neurodegeneration
MWM: Morris Water Maze
PNN: Perineuronal net
snRNA-seq: Single-nucleus RNA sequencing
Trem2: Triggering receptor expressed on myeloid cells 2
UMAP: Uniform Manifold Approximation and Projection
