# Supplementary material for: Insulin resistance alters cortical inhibitory neurons and microglia to exacerbate Alzheimer’s knock-in mouse phenotypes
Source: Mol Neurodegener. 2026 Jun 3;21:44. doi: 10.1186/s13024-026-00946-0 (PMC13459496; doi:10.1186/s13024-026-00946-0)
Supplement: Supplementary file 1 [file 13024_2026_946_MOESM1_ESM.docx]

**Supplementary Materials**

**Supplementary Fig. S1: DKI-HFHS mice experience deficits in learning, but not memory consolidation**

**a-c,** Reverse learning performance in the MWM. **a-b,** Latency time to the hidden platform during the reverse training phase (**a**) and final training session (**b**). **c,** Fraction of time spent in the target quadrant, during reverse probe trials. (For **a-c,** *n=14* for WT-Lean and WT-HFHS groups; *n=15* for DKI-Lean and DKI-HFHS groups). Data are presented as mean ± SEM and analyzed by two-way ANOVA with Dunnett’s multiple comparison test (**a**) or one-way ANOVA with Tukey’s multiple comparisons test (**b,c**), **p<*0.0332; ns, not significant.

**Supplementary Fig. S2: Filament tracing and Sholl analysis of microglial complexity**

**a-e**, Assessment of microglial morphology using filament tracing in Imaris. **a**, **Representative 3D renderings of microglia morphologies generated in Imaris (top; scale bars: 15** µm**) and corresponding filament tracings of the boxed region (bottom; scale bars: 5** µm**).** Somas are shown in blue, branches in red, and terminal points in green. **b**, Quantification of the number of microglia filaments at a radial distance of 10 μm from the soma. **c-e,** Quantification of total process length (**c**), number of terminal points (**d**), and number of branch points (**e**). Data are presented as mean ± SEM and analyzed by one-way ANOVA with Tukey’s multiple comparisons test, **p<*0.0332, ***p<*0.0021, ****p<*0.0002, and ****p<0.0001; ns, not significant.

**Supplementary Fig. S3: Aβ pathology and microglial plaque association are not impacted by HFHS diet**

**a-d**, Assessment of microglial morphology and plaque associations using volumetric reconstructions in Imaris. **a**, Right: Representative 3D renderings of microglial morphology (Iba1, green) and amyloid beta deposition (red). Left: Example Imaris-generated surface models of microglia (magenta) and amyloid plaques (colored by scaled volume) for DKI groups used in subsequent analyses in **b-d**. Scale bars, 15 µm. **b,** quantification Aβ deposition area shown in **a**. **c,** Distribution of amyloid beta plaque volumes for cell quantified in **b**. **d,** Frequency of microglia-plaque associations for cells quantified in **b**.

**Supplementary Fig. S4: HFHS-diet induced synaptic deficits occur independently of tau pathology in DKI-mice**

**a-d, Image analysis of Tau makers AT8 and pThr217 in the mouse cortex. a.** Representative images of anti-Iba1 (white), anti-Aβ (red), and anti-AT8 (green) immunostaining across treatment groups. **b,** Quantification of AT8 percent area represented in **a.** Each data point represents the mean value of three images per mouse. **c,** Representative images of anti-Trem2 (red), anti-**pThr217** (green) immunostaining. **d,** Quantification of **pThr217** percent area represented in **c**. Each data point represents the mean value of 4-6 images per mouse. (For **b,d,** *n=9* for WT-Lean group; *n=12* for WT-HFHS, *n=11* for DKI-Lean, and *n=10* for DKI-HFHS groups). For **b,d,** Data presented as the mean ± SEM and analyzed by one-way ANOVA with Kruskal-Wallis test with Dunn’s multiple comparisons corrections, **p<*0.0332, and ***p<*0.0021, ****p<*0.0002; ns: not significant. **e-f,** Representative staining of pre-synaptic anti-Synapsin1/2 (green) and post-synaptic PSD-95 (magenta) colocalization within cortical region L2 (**e**), synaptic quantification (**f**). (*n=6,* WT-Lean; *n=10*, WT-HFHS; *n=10,* DKI-Lean; and *n=11,* DKI-HFHS groups). For **f,** data presented as the mean ± SEM and analyzed by two-way ANOVA with Tukey’s multiple comparisons test, **p<*0.0332 and ***p<*0.0021. For **b,d,f,** scale bars, 10 μm.

**Supplementary Fig. S5: Single-nucleus RNA-seq reveals prominent transcriptional shifts in glial and inhibitory neuron populations**

**a-g,** Integration and cell type classification of snRNA-seq datasets. **a,** UMAP embedding of single-nucleus transcriptomes showing cellular clustering based on gene expression profiles. Each point represents a single nucleus colored by annotated cell types shown in **b**. **b,** Dot plot showing the percentage of nuclei and scaled mean expression levels of cell-type classification markers corresponding to clusters shown in **a**. **c,** Mean nuclei frequency of cell types across individual samples by genotype, separated into neuronal (top) and non-neuronal (bottom) populations. Data are presented as mean ± SEM and analyzed by two-way ANOVA test with Tukey’s multiple comparisons correction. (*n=4* per group, **p<*0.0332, ***p<*0.0021, ****p<*0.0002, and ****p<0.0001)*.* **d-f,** Quality control metrics per sample, including total nuclei (**d**), mean reads per nucleus (**e**), and mean number of unique genes detected per nucleus (**f**). **g,** Group-specific expression profiles projected onto the UMAP shown in **a**. Notable transcriptomic shifts are observed within microglial clusters (closed arrows) and a subset of inhibitory neuron nuclei (open arrow). See Supplemental Tables 1 and 2 for cluster marker genes and cluster cell type classification. snRNAseq, single-nuclei RNA sequencing; UMAP, Uniform Manifold Approximation and Projection; Ex. Neurons, excitatory neuron; In. Neurons, inhibitory neuron; OPCs, oligodendrocyte precursor cells; OligoD, oligodendrocytes; CPEs, ciliary pigmented epithelial cells (ependymal cells); ABCs, arachnoid barrier cells; vLMCs, vascular leptomeningeal cells.

**Supplementary Fig. S6:** **Disease-activated microglial signatures show HFHS-modulated expression in DKI mice**

**a-b,** UMAP of microglia subclusters (**a**), and individual group expression profiles (**b**). **c,** Transcript levels of gene markers representing microglia homeostatic, disease activation, and MinD physiological states in UMAP space are shown in **a-b**. **d,** Comparative heatmaps showing the relative expression patterns of top genes representing curated microglia cell-state markers [1], restricted to mouse genes and mouse orthologues of human genes. **e,** Venn diagrams depicting the intersection between microglial state gene signatures shown in **d** and total DKI-HFHS DEGs (vs WT-Lean), demonstrating that the MinD program represents a smaller, distinct subset. Data related to Fig. 3. MinD: Metabolic impairment in neurodegeneration; HM: homeostatic microglia, HLA: human leukocyte antigen, CRM: cytokine response microglia; IRM/IFN; Interferon Response Microglia.

**Supplementary Fig. S7: HFHS diet induces a MinD state in astrocyte transcriptomes of DKI mice**

**a**, Representative images of cortical astrocyte coverage (red) and amyloid-β deposition (green). **b**, Quantification of astrocyte coverage represented by GFAP signal in (**a**). Data are presented as mean ± SEM and analyzed by one-way ANOVA test with Tukey’s multiple comparisons correction. (*n=9,* WT-Lean; *n=11*, for WT-HFHS*,* DKI-Lean, and DKI-HFHS groups. ****p<0.0001; ns: not significant). **c-g**, Transcriptomic assessment of astrocyte changes in response to diet, disease, and diet-disease interaction. **c,** UMAP of astrocyte subcluster of overlayed (left) and individual (right) group expression profiles. **d,** Venn diagram showing shared and unique DEGs in astrocytes between WT-HFHS, DKI-Lean, and DKI-HFHS groups as compared to WT-Lean. Of the DKI-HFHS DEGS, 122 genes are unique to the MinD transcriptional state. **e,** Venn diagram showing that MinD-specific DEGs are largely distinct from published DAA cell state markers [2], with only 7 overlapping DEGs. **f,** Nuclei transcript levels of the top six marker genes for DKI-HFHS astrocytes. **g,** Correlation plot comparing Log_2_ fold-changes of DEGs in DKI-Lean and DKI-HFHS astrocyte groups relative to WT-Lean. See Supplemental Table 4 for the complete DEG list. GFAP: [Glial fibrillary acidic protein](https://www.google.com/search?q=Glial+fibrillary+acidic+protein&rlz=1C5CHFA_enUS953US953&oq=GFAP&gs_lcrp=EgZjaHJvbWUqDQgAEAAYgwEYsQMYgAQyDQgAEAAYgwEYsQMYgAQyBwgBEAAYgAQyBwgCEAAYgAQyBwgDEAAYgAQyBwgEEAAYgAQyBwgFEAAYgAQyBwgGEAAYgAQyBwgHEAAYgAQyBwgIEAAYgAQyBwgJEAAYgATSAQkyNDMxajBqMTWoAgiwAgHxBfvLiqi80t7E&sourceid=chrome&ie=UTF-8&ved=2ahUKEwjt6KGB5JWTAxUxAHkGHan8A5IQgK4QegYIAQgAEAQ); DEGs: differentially expressed genes; DAA: Disease-associated astrocytes, CPM: counts per million.

**Supplementary Fig. S8: HFHS diet induces a MinD state in oligodendrocyte transcriptomes of DKI mice**

**a-f**, Transcriptomics profiles of oligodendrocytes in response to diet, disease, and diet-disease interaction. **a-b**, UMAP of OPCs and oligodendrocytes subclusters of overlayed (**a**) and individual (**b**) group expression profiles. **c**, Venn diagram showing shared and unique DEGs in oligodendrocytes between WT-HFHS, DKI-Lean, and DKI-HFHS groups as compared to WT-Lean. **d,** Correlation plot comparing Log_2_ fold-changes of DEGs in DKI-Lean and DKI-HFHS oligodendrocytes relative to the WT-Lean group. **e,** Nuclei transcript levels of the top six marker genes for DKI-HFHS oligodendrocytes. See Supplemental Table 4 for the complete DEG list. ns: not significant; DEGs: differentially expressed genes; CPM: counts per million.

**Supplementary Fig. S9: HFHS and disease induce changes in perineuronal nets in WT and DKI mice**

**a,** Representative images showing WFA (red) and anti-Pvalb (Green) staining. Scale bars, 100 μm. **b,c,** Quantification of the percent area of WFA coverage (**b**) and Pvalb^+^ cells (**c**). (*n=9* for WT-Lean and WT-HFHS groups; *n=10* for DKI-Lean and DKI-HFHS groups). **c,d,** Data presented as the mean ± SEM and analyzed by two-way ANOVA with Tukey’s multiple comparisons test, ***p<*0.002; ns: not significant.

**Supplementary Fig. S10: Body weight corresponds to diet nutrient composition**

Body weight measurements were taken at 11.5 months of age in WT and DKI mice on Standard, Lean, or HFHS diet. The number of mice per group is displayed within each bar. Standard-diet mice were fed *ad libitum* at weaning*.* Mice on Lean or HFHS diets were fed *ad libitum* as described in the methods. Data are presented as mean ± SEM and analyzed by one-way ANOVA with Tukey’s multiple comparisons test. Only *p-values* for means with significant differences are shown, **p<*0.0332, ***p<*0.0021, ****p<*0.0002, and ****p<0.0001.

**References**

1.	Fumagalli L, Nazlie Mohebiany A, Premereur J, Polanco Miquel P, Bijnens B, Van de Walle P, et al. Microglia heterogeneity, modeling and cell-state annotation in development and neurodegeneration. Nat Neurosci. 2025.

2.	Habib N, McCabe C, Medina S, Varshavsky M, Kitsberg D, Dvir-Szternfeld R, et al. Disease-associated astrocytes in Alzheimer's disease and aging. Nat Neurosci. 2020;23(6):701-6.

**Supplementary Tables**

**Supplementary Table 1.** Gene expression marker list. Marker genes were determined using *Scanpy’s rank_gene_groups* function, applying the Wilcoxon rank-sum test, with adjustment for multiple comparisons via the Benjamini-Hochberg method.

**Supplementary Table 2.** Cell-type classifications as determined by the Allen Brain Atlas MapMyCells tool (RRID:SCR_024672).

**Supplementary Table 3.** Differentially expressed genes and pathway enrichment analysis for microglia.

**Supplementary Table 4.** Differentially expressed genes in astrocytes and oligodendrocytes.

**Supplementary Table 5.** Differentially expressed genes and pathway enrichment analysis for Meis2-positive inhibitory neurons.

**Supplementary Table 6.** Differentially expressed genes and pathway enrichment analysis for ExN 10 subcluster of excitatory neuron nuclei representing cortical layers 2-4

**Supplementary Fig. S1**

**Supplementary Fig. S2**

**Supplementary Fig. S3**

**Supplementary Fig. S4**

**Supplementary Fig. S5**

**Supplementary Fig. S6**

**Supplementary Fig. S7**

**Supplementary Fig. S8**

**Supplementary Fig. S9**

**Supplementary Fig. S10**
